# Supplementary material for: Low fucosylation defines the glycocalyx of progenitor cells and melanocytes in the human limbal stem cell niche
Source: Stem Cell Reports. 2024 Dec 19;20(1):102378. doi: 10.1016/j.stemcr.2024.11.008 (PMC11784483; doi:10.1016/j.stemcr.2024.11.008)
Supplement: Document S1. Figures S1–S4 and Supplemental experimental procedures [file mmc1.pdf]

Stem Cell Reports, Volume *20*

Supplemental Information

Low fucosylation defines the glycocalyx of progenitor cells and melanocytes in the human limbal stem cell niche

Ashley M. Woodward, Damien Guindolet, Rafael Martinez-Carrasco, Eric E. Gabison, Robert M. Lavker, and Pablo Argüeso

Supplemental Figures

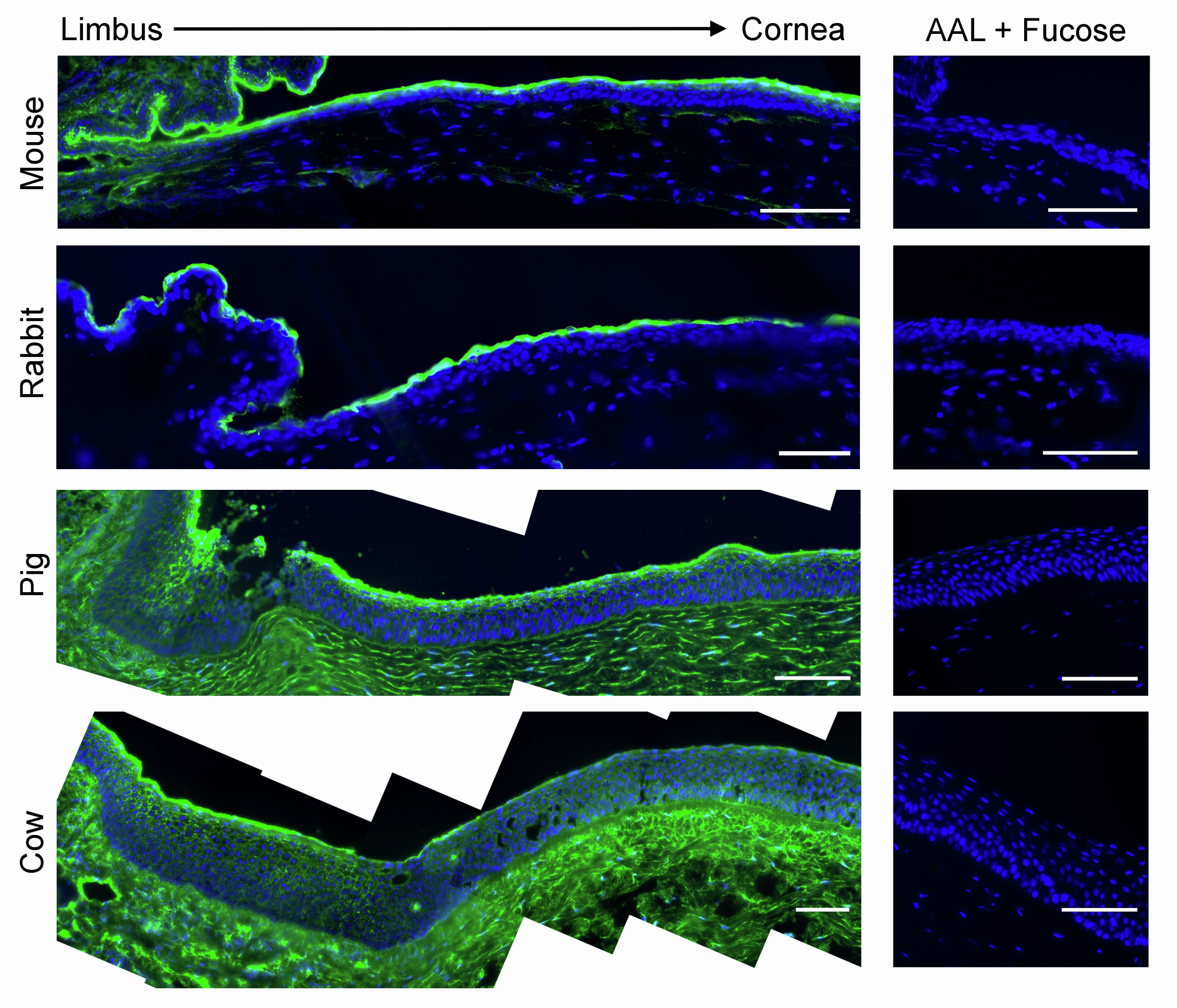

Supplemental Figure S1.

Representative images showing AAL histochemistry (green) and DAPI (blue) in limbus and cornea of different adult mammalian species. AAL binding was inhibited in the presence of competing L-fucose. Scale bars, 100 μm.

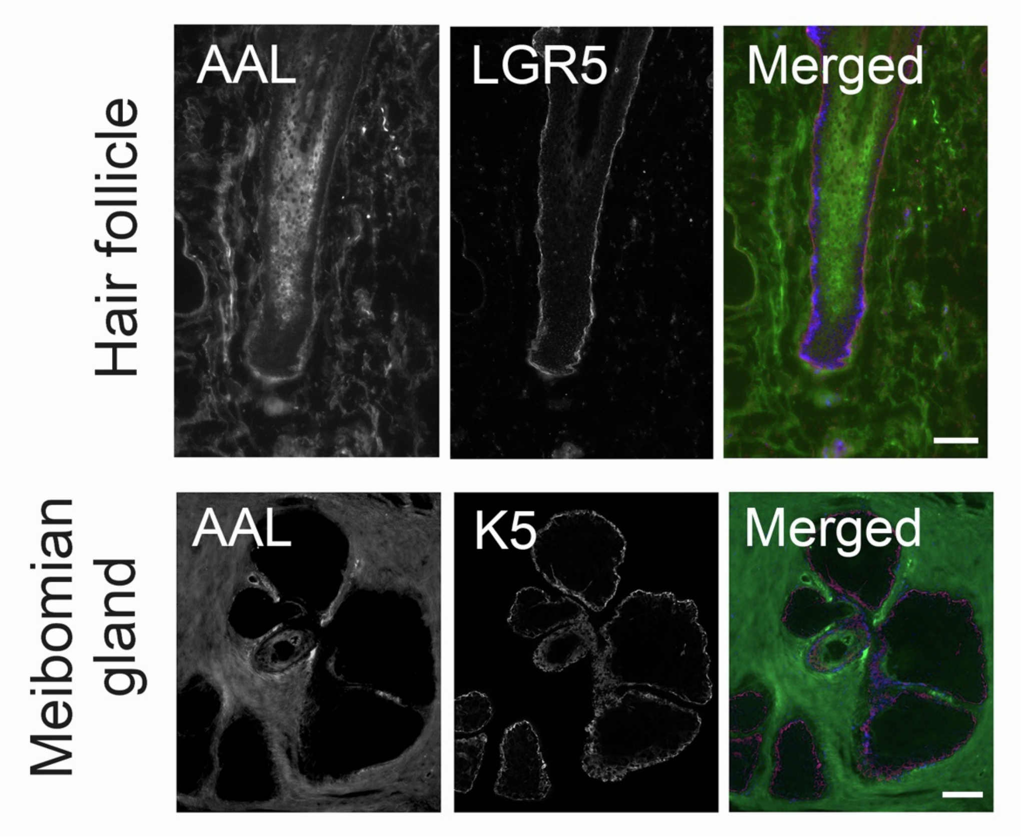

Supplemental Figure S2.

Representative costaining images of AAL (green) and LGR5 (red) in the hair follicle, or cytokeratin 5 (red) in the meibomian gland. Nuclear DNA in the merged image was stained with DAPI (blue). Scale bars, 100 μm.

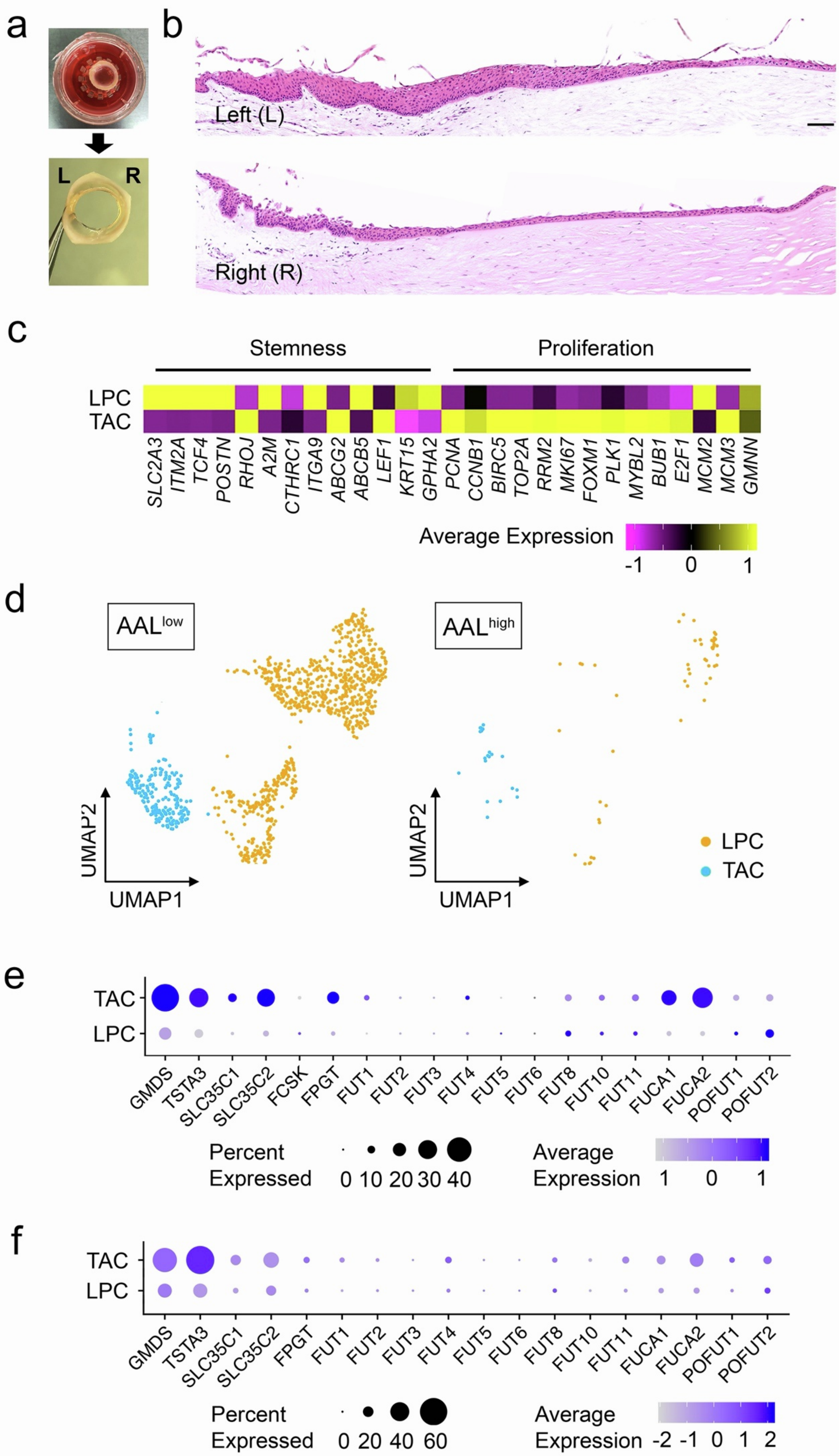

a
L
R
b
Left (L)
Right (R)
c
Stemness
Proliferation
LPC
TAC
SLC2A3
ITM2A
TCF4
POSTN
RHOJ
A2M
CTHRC1
ITGA9
ABCG2
ABCB5
LEF1
KRT15
GPHA2
PCNA
CCNB1
BIRC5
TOP2A
RRM2
MKI67
FOXM1
PLK1
MYBL2
BUB1
E2F1
MCM2
MCM3
GMNN
Average Expression
-1 0 1
d
AAL^low
AAL^high
UMAP2
UMAP1
LPC
TAC
e
TAC
LPC
GMDS
TSTA3
SLC35C1
SLC35C2
FCSK
FPGT
FUT1
FUT2
FUT3
FUT4
FUT5
FUT6
FUT8
FUT10
FUT11
FUCA1
FUCA2
POFUT1
POFUT2
Percent Expressed
0 10 20 30 40
Average Expression
1 0 1
f
TAC
LPC
GMDS
TSTA3
SLC35C1
SLC35C2
FPGT
FUT1
FUT2
FUT3
FUT4
FUT5
FUT6
FUT8
FUT10
FUT11
FUCA1
FUCA2
POFUT1
POFUT2
Percent Expressed
0 20 40 60
Average Expression
-2 -1 0 1 2

Supplemental Figure S3.

(a) Isolation of the limbal region of the cornea. The outer ring contains peripheral cornea, limbus and adjacent conjunctiva. L, left portion; R, right portion.

(b) Histological assessment of peripheral corneal donor tissue (left and right portions) by hematoxylin and eosin staining. Scale bar = 100 μm.

(c) Heatmap showing expression patterns of genes related to stemness and proliferation in limbal progenitor (LPC) and transient amplifying (TAC) cell subpopulations, identified by the reclustering of corneal epithelial cells, as described (Li et al., 2021).

(d) UMAP visualization of LPC and TAC cell subpopulations within the AAL^{low} and AAL^{high} fractions.

(e) The dot plot depicts expression levels of fucosylation genes in the LPC and TAC cell subpopulations together with the percentage of cells expressing each gene in our datasets.

(f) The dot plot depicts expression levels of fucosylation genes in published LPC and TAC cell subpopulations together with the percentage of cells expressing each gene in a database from a public repository (data accessible at NCBI GEO database, accession GSE153515) (Li et al., 2021).

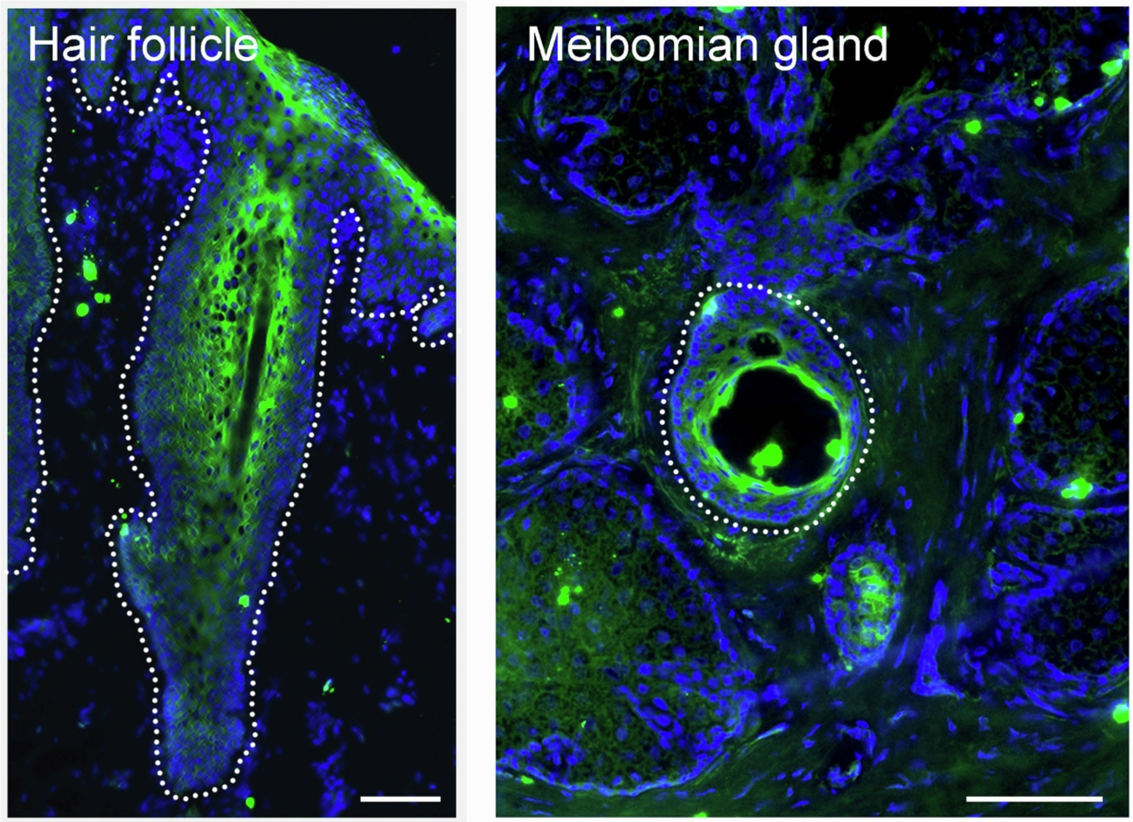

Supplemental Figure S4.

Representative micrographs showing GMDS distribution (green) in human hair follicle and meibomian gland specimens. Nuclear DNA was stained with DAPI (blue). Dashed lines denote the epithelial-stromal junction. Scale bars, 100 μm.

Supplemental Experimental Procedures

Tissue source

All procedures on human tissue were performed in accordance with the Declaration of Helsinki and approved by the Health Sciences Institutional Review Board at Tufts University. Human postmortem corneoscleral tissue unsuitable for transplantation was obtained from Lions VisionGift (Portland, OR) and Saving Sight (Kansas City, MO). Standard eye bank protocols for informed consent and protection of donor confidentiality were used. Standard serologies for infectious agents were negative for all donors. The mean age of the donors (5 females, 5 males) was 61.3 ± 3.8 years (range, 50 to 88 years). The average time from death to preservation of corneoscleral tissue was 14.0 ± 1.4 hours and the average time the tissue was kept in organ culture was 4.6 ± 0.5 days. Archived human eyelid tissue removed during lid resection surgery was obtained from a previous study (Ubels et al., 2012). Eyes from other animal species (cow, pig, rabbit and mouse) were obtained immediately after death from a local slaughterhouse or animal facility.

Preparation of corneal and limbal epithelial cell suspensions

The iris root and endothelium were scraped from corneoscleral tissue using a K-sponge spear (Katena). The central portion of the tissue was cut with an 8-mm disposable biopsy punch (Integra Miltex) to produce corneal rims. Corneal rims and central cornea were subsequently incubated for 1 hour at 37 °C with 2.4 IU/mL Dispase II (Thermo Fisher Scientific) in a supplemented growth medium (sGMedium) containing a 3:1 mixture of DMEM GlutaMAX:F12 with 10% fetal bovine serum, 0.4 μg/mL hydrocortisone, 5 μg/mL insulin, 1.4 ng/mL triiodothyronine, 24 μg/mL adenine, 8.4 ng/mL cholera toxin, 10 ng/mL epidermal growth factor and 1% antibiotic–antimycotic. The epithelia were scraped with a sterile disposable scalpel no. 10 (Integra Miltex), centrifuged at 400 × g for 5 minutes and resuspended in TrypLE Express for another 5 minutes. Cells were then washed with DPBS lacking calcium and magnesium and filtered through a 35 μm nylon cell strainer (Corning).

Growth-arrested 3T3-J2 cells

3T3-J2 mouse fibroblasts (Kerafast) were maintained in DMEM GlutaMAX with 10% bovine calf serum (Hyclone) and 1% penicillin/streptomycin in a humidified incubator with 5% CO_2. Fibroblasts were mitotically inactivated by incubation with 4 μg/mL mitomycin-C (Sigma-Aldrich) for 2 hours at 37 °C. Cells were then rinsed five times with DPBS lacking calcium and magnesium, detached with TrypLE Express (Thermo Fisher Scientific) and frozen in DMEM

GlutaMAX supplemented with 20% bovine calf serum and 10% dimethylsulfoxide (DMSO) for 2 hours at -80 °C before cryopreservation in liquid nitrogen.

Ex vivo expansion and culture of human limbal epithelial cells

Growth-arrested 3T3-J2 fibroblasts were suspended in DMEM GlutaMAX with 10% fetal bovine serum and plated at a density of 4.6 × 10^4 cells/cm^2 in a 100-mm tissue culture dish (Corning). On the following day, limbal epithelial cell suspensions were resuspended in sGMedium and seeded at a density of up to 6 × 10^3 cells/cm^2 in 100-mm tissue culture dishes containing growth-arrested 3T3-J2 fibroblasts. The sGMedium was changed on the third day and every other day thereafter. After 7 days, the 3T3-J2 feeder cells were detached using Versene (Thermo Fisher Scientific) for approximately 1 minute, followed by a wash in DPBS and a 10-minute incubation with TrypLE Express to detach the epithelial cells. These cells were pelleted by centrifugation and frozen (passage 0; cell bank) in DMEM GlutaMAX supplemented with 20% fetal bovine serum and 10% DMSO.

Viable epithelial cells, as determined using Trypan Blue exclusion, were plated at a density of 2 × 10^4 cells/cm^2 for lectin histochemistry or 1 × 10^5 cells/cm^2 for immunoblot and lectin blot. Epithelial cells used for lectin histochemistry, or lectin blot, were grown in the presence of growth-arrested fibroblasts. The fibroblasts were detached for imaging or before protein was collected from the epithelial cells. Immunoblot analyses were performed using protein from epithelial cells grown alone, as previously described (Guindolet et al., 2022). For fucosylation inhibition assays, cells were incubated continuously with 100 μM 2F-peracetyl-fucose (Sigma-Aldrich) or vehicle control (DMSO).

Culture of human limbal melanocytes

Human limbal melanocytes were cultured as previously described (Polisetti et al., 2020). Briefly, 2-10 × 10^5 cells harvested from corneoscleral tissues were seeded onto T75 tissue culture flasks and grown in Medium 254 supplemented with HGMS-2 (Thermo Fisher Scientific). Medium was changed on the first day and every other day thereafter. After 10-14 days, melanocytes and fibroblasts were enzymatically isolated from epithelial cells based on differences in substrate adherence by incubation with a 0.025% trypsin-0.01% EDTA solution (Thermo Fisher Scientific) for 1-3 minutes at 37 °C. After centrifugation, cells were resuspended in Medium 254 supplemented with HGMS-2 and replated at a density of 5 × 10^3 cells/cm^2. Upon reaching 80% confluence, cultures were treated with 0.2 mg/ml geneticin (Thermo Fisher Scientific), an inhibitor of protein synthesis that differentially affects the viability of melanocytes and fibroblasts, for 48 hours to inhibit the growth of fibroblasts. Geneticin was added up to three times to achieve

a yield of >95% melanocytes before expansion in CnT-40 medium (Zen-Bio). Melanocytes were plated onto Millicell EZ 8-well glass chamber slides at a density of 2×10^4 cells/cm^2.

For evaluation of melanocyte enrichment, single cell suspensions of geneticin treated cells were incubated with FcR blocking reagent (20 μl/10^6 cells; Miltenyi Biotec) for 5 minutes and washed with PBS. Cells were then incubated with APC-conjugated anti-CD117 antibody (A3C6E2; Miltenyi Biotec) or isotype control antibody (10 μl/10^6 cells) in PBS containing 0.1% sodium azide and 2% fetal bovine serum for 30 minutes at 4 °C in the dark. After washing with PBS, cells were analyzed with a BD LSR II flow cytometer (BD Pharmingen).

Supplemental References

Guindolet, D., Woodward, A.M., Gabison, E.E., and Argueso, P. (2022). Glycogene expression profile of human limbal epithelial cells with distinct clonogenic potential. Cells *11*. 10.3390/cells11091575.

Li, D.Q., Kim, S., Li, J.M., Gao, Q., Choi, J., Bian, F., Hu, J., Zhang, Y., Li, J., Lu, R., et al. (2021). Single-cell transcriptomics identifies limbal stem cell population and cell types mapping its differentiation trajectory in limbal basal epithelium of human cornea. Ocul Surf *20*, 20-32. 10.1016/j.jtos.2020.12.004.

Polisetti, N., Gießl, A., Li, S., Sorokin, L., Kruse, F.E., and Schlötzer-Schrehardt, U. (2020). Laminin-511-E8 promotes efficient in vitro expansion of human limbal melanocytes. Sci Rep *10*, 11074. 10.1038/s41598-020-68120-0.

Ubels, J.L., Gipson, I.K., Spurr-Michaud, S.J., Tisdale, A.S., Van Dyken, R.E., and Hatton, M.P. (2012). Gene expression in human accessory lacrimal glands of Wolfring. Invest Ophthalmol Vis Sci *53*, 6738-6747. 10.1167/iovs.12-10750.
