# Supplementary material for: Low fucosylation defines the glycocalyx of progenitor cells and melanocytes in the human limbal stem cell niche
Source: Stem Cell Reports. 2024 Dec 19;20(1):102378. doi: 10.1016/j.stemcr.2024.11.008 (PMC11784483; doi:10.1016/j.stemcr.2024.11.008)
Supplement: Document S2. Article plus supplemental information [file mmc2.pdf]

Stem Cell Reports
Report
OPEN ACCESS

Low fucosylation defines the glycocalyx of progenitor cells and melanocytes in the human limbal stem cell niche

Ashley M. Woodward,[1] Damien Guindolet,[2] Rafael Martinez-Carrasco,[1] Eric E. Gabison,[2] Robert M. Lavker,[3] and Pablo Argüeso[1,*]
[1]Department of Ophthalmology, Tufts Medical Center, Tufts University School of Medicine, 150 Harrison Avenue, Boston, MA 02111, USA
[2]Fondation Ophtalmologique A. de Rothschild, 25 rue Manin, 75019 Paris, France
[3]Department of Dermatology, Northwestern University, 303 East Chicago Avenue, Chicago, IL 60611, USA
*Correspondence: pablo.argueso@tufts.edu
https://doi.org/10.1016/j.stemcr.2024.11.008

SUMMARY

It is widely recognized that the glycocalyx has significant implications in regulating the self-renewal and differentiation of adult stem cells; however, its composition remains poorly understood. Here, we show that the fucose-binding *Aleuria aurantia* lectin (AAL) binds differentially to basal cells in the stratified epithelium of the human limbus, hair follicle epithelium, and meibomian gland duct. Using fluorescence-activated cell sorting in combination with single-cell transcriptomics, we find that most epithelial progenitor cells and melanocytes in the limbus display low AAL staining (AAL^{low}) on their cell surface, an attribute that is gradually lost in epithelial cells as they differentiate into mature corneal cells. AAL^{low} epithelial cells were enriched in putative limbal stem cell markers and displayed high clonogenic capacity. Further analyses revealed that AAL^{low} epithelial cells had reduced expression of GDP-mannose-4,6-dehydratase, an enzyme catalyzing the first and limiting step in the *de novo* biosynthesis of GDP-fucose, and that inhibition of fucosylation using a small-molecule fucose analog stimulated the proliferative potential of limbal epithelial cells *ex vivo*. These results provide crucial insights into the distinctive composition of the glycocalyx in adult stem cells and underscore the significance of fucose modulation in the therapeutic regeneration of the human limbal stem cell niche.

INTRODUCTION

Adult stem cells, also known as somatic or nonembryonic stem cells, are undifferentiated cells located within specialized niches in the adult organism. They are responsible for maintaining normal tissue turnover and mounting a regenerative response following acute injury (de Morree and Rando, 2023). These cells are characterized by a high capacity of self-renewal, slow cell cycle, and ability to produce daughter cells that undergo proliferation and then differentiation. Populations of adult stem cells have been identified within multiple tissue types. In the corneal epithelium, they localize to the basal layer at the transition zone between the peripheral cornea and the anterior sclera, in an area known as the limbus (Figure 1A) (Cotsarelis et al., 1989). From this location, limbal epithelial stem cells give rise to transient amplifying cells that migrate centripetally and anteriorly to generate the mature corneal epithelium. The limbal stem cell niche contains a distinctive basement membrane and is enriched with various cell populations, such as melanocytes and immune cells. Multiple lines of evidence indicate that this unique microenvironment is critical to the prevention of stem cell differentiation and defining stem cell fate (Gesteira et al., 2017; Gonzalez et al., 2018; Secker and Daniels, 2009).

Uncovering the molecular signatures of adult stem cells as they transition from a self-renewing state into a specialized mature tissue remains a fundamental objective in stem cell biology. This is a highly regulated process thought to be orchestrated primarily by the activation of specific transcriptional pathways and epigenetic modifications (Avgustinova and Benitah, 2016). Recent studies are highlighting the contribution of additional signals, such as those driven by metabolic cues, in the regulation of cell fate decisions (Tatapudy et al., 2017). Carbohydrates found on glycoproteins and glycolipids in the glycocalyx also play decisive roles in the control of cell fate, by modulating intracellular cell signaling and the interactions of cells with the microenvironment (Furukawa et al., 2012). The composition of the glycocalyx changes during differentiation and has been used for identifying and isolating specific cell types from heterogeneous populations (Lanctot et al., 2007). Research during the past decade has started to uncover the potential of altering the glycocalyx in the adult stem cell niche for therapeutic gain. One example is the enzymatic modification of N-acetyllactosamine on the surface of murine Paneth cells, which promotes hyperproliferation of neighboring stem cells in intestinal crypt organoids (Rouhanifard et al., 2018). Yet, progress in this field has been hindered by a shortage of details on the glycans present in the glycocalyx of stem cells across adult tissues and their relevance to maintaining self-renewal and differentiation capabilities.

The formation of the glycocalyx involves the coordinated action of metabolic enzymes, nucleotide sugar transporters, hydrolases, and multiple glycosyltransferases that

© 2024 The Author(s).
Published by Elsevier Inc. on behalf of International Society for Stem Cell Research.
This is an open access article under the CC BY license (http://creativecommons.org/licenses/by/4.0/).

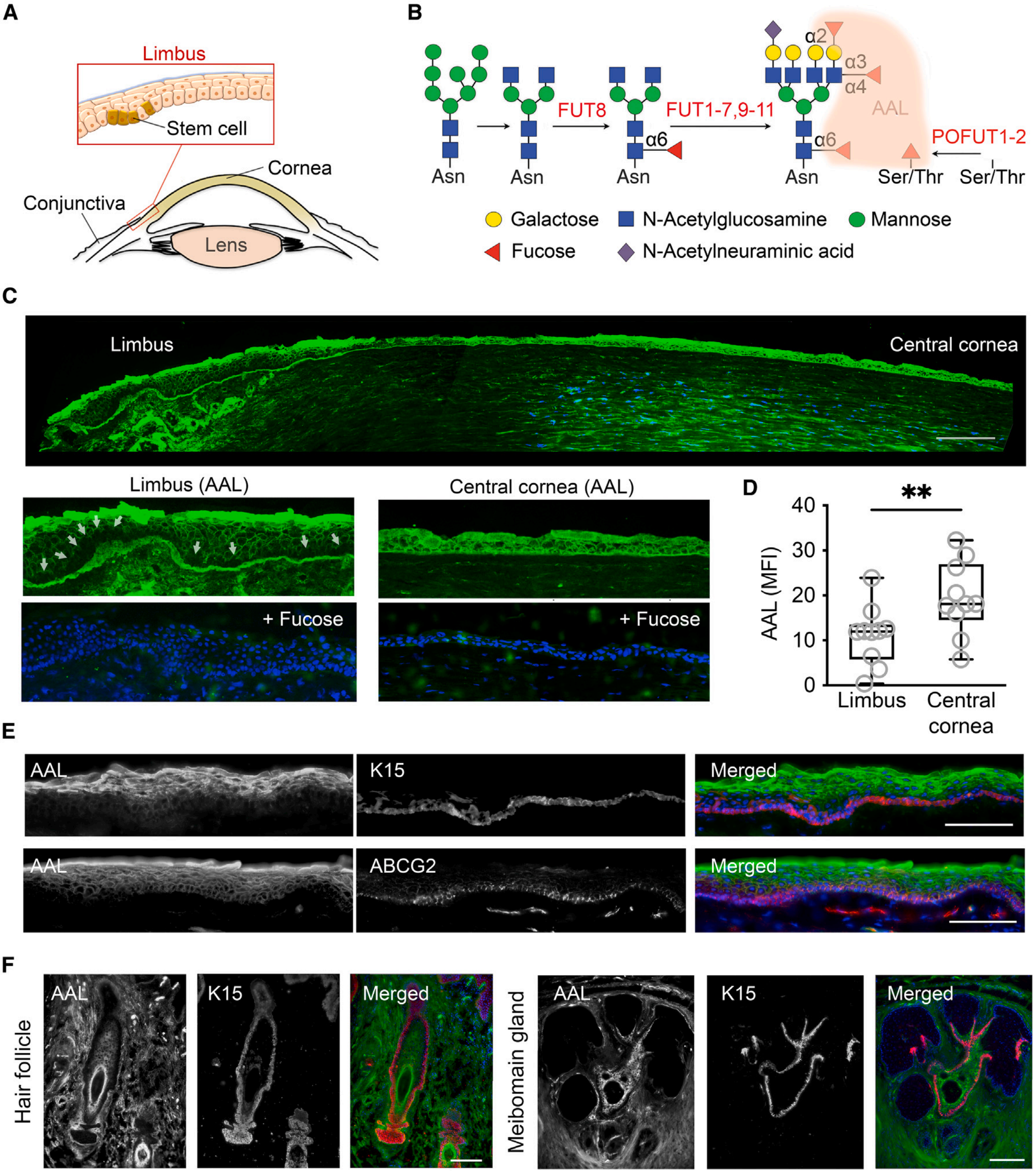

Figure 1. AAL binds differentially to basal cells in human stratified epithelia

(A) Schematic representation of adult stem cells located in the basal epithelial layer of the human limbus.

(B) Simplified diagram showing the post-translational modification of proteins by fucosyltransferases (FUT1-11, POFUT1-2) and the interactions between AAL and fucose residues.

(C) Representative image showing AAL histochemistry (green) and DAPI (blue) staining in the human cornea (top) of five human donors. Bottom images show magnified images from the same corneal section. Arrows indicate pockets of cells with negligible AAL staining. AAL binding was inhibited in the presence of competing L-fucose. Scale bar, 100 μm.

(D) Quantification of AAL staining across the basal layer of the human limbal and central corneal epithelia. Two independent sections were analyzed from each donor. The box-and-whisker plots show the mean fluorescence intensity, MFI ($n = 5$ human donors; $^{**}p < 0.01$, paired t test).

(E) Immunohistochemical analysis of AAL (green) co-stained with K15 or ABCG2 (red) in the human limbus. Scale bars, 100 μm.

(F) Immunohistochemical analysis of AAL (green) co-stained with K15 (red) in human hair follicle and meibomian gland specimens. Scale bars, 100 μm.

work together to synthesize and modify glycan chains (Scheper et al., 2023). In mammals, fucose-containing glycan chains have important roles in development and the regulation of signal transduction (Becker and Lowe, 2003). The addition of fucose to glycoconjugates requires the synthesis of a nucleotide-activated form of fucose, GDP-fucose, in the cytoplasm, which occurs primarily via the *de novo* pathway (Skurska et al., 2022). GDP-D-mannose-4,6-dehydratase (GMDS) is the first enzyme, and limiting step, in this pathway (Bisso et al., 1999). It catalyzes the conversion of GDP-D-mannose into the GDP-4-keto-6-deoxy-D-mannose intermediate, which is then epimerized and finally reduced to produce GDP-fucose (Sullivan et al., 1998). Crucial experiments in the *Drosophila* intestine have shown that loss of this enzyme does not influence terminal differentiation but leads to aberrant self-renewing stem cell divisions that generate increased numbers of stem cells (Perdigoto et al., 2011). Yet, the implications of these findings to mammalian organisms, and humans in particular, remain unknown. In the present study, we demonstrate that the fucose-binding *Aleuria aurantia* lectin (AAL) displays weak binding to progenitor cells within three human epithelial compartments. Moreover, we find that limbal epithelial cells with low fucosylation levels are enriched in putative limbal stem cell markers and display high clonogenic capacity. Further experiments revealed that these cells produce low levels of GMDS and uncovered the potential of inhibiting fucosylation to stimulate the proliferative potential of limbal epithelial cells *ex vivo*.

RESULTS

AAL binds differentially to basal cells in human stratified epithelia

AAL is a lectin derived from the fungus *Aleuria aurantia* well-known for its ability to bind fucose-containing glycans (Figure 1B). AAL has high specificity for α-linked fucose synthesized by fucosyltransferases (FUT1-11) and protein O-fucosyltransferases (POFUT1-2) located throughout the secretory pathway of eukaryotic cells (Bandini et al., 2016; Bojar et al., 2022). Analysis of AAL distribution in the human limbus revealed membranous staining along the suprabasal epithelial cell layers, with the most intense signal detected within the superficial cells (Figure 1C). Remarkably, we found that the basal layer of the limbus was defined by a patchy distribution of the lectin and the presence of pockets of cells with negligible staining. Conversely, basal cells of the central corneal epithelium exhibited a more intense and homogeneous pattern of staining compared to the basal limbal epithelium (Figures 1C and 1D). In co-staining experiments, we observed a relationship between the absence of AAL staining and the presence of cytokeratin 15 (K15) and ABCG2, two putative markers of limbal stem cells (Figure 1E). Further examination of AAL staining in other mammalian tissues revealed differences across species (Figure S1). AAL staining in mice and rabbits was predominant in terminally differentiated superficial cells and low or absent in basal and suprabasal cells across the entire corneal epithelium. Pigs and cows, on the other hand, showed a gradual decrease of staining toward the basal layer of the limbus, similarly to humans. In these experiments, AAL binding could be inhibited by preincubation with L-fucose, indicating the specificity of the carbohydrate-lectin interaction.

In additional experiments, we asked whether the differential pattern of AAL staining in the limbus could be identified in other human stratified epithelia. Hair follicles contain a clearly defined anatomical region called the bulge, which preferentially expresses K15 and LGR5 and contains adult stem cells (Lyle et al., 1998; Polkoff et al., 2022). We found a negative correlation between the localization of these markers and AAL in the hair follicle, with K15 and LRG5 being primarily present in the basal layer of the epithelium and AAL showing increased staining toward the more differentiated cells of the hair shaft (Figures 1F and S2). Similar results were observed when evaluating the human meibomian gland. Here, the quiescent progenitor cells are located across the basal layer of the epithelium, including the terminal ends of the ductal epithelium where it meets the secretory acini (Parfitt et al., 2016). The expression of the putative stem cell markers K15 and K5 was observed along the basal layer of the epithelium, in clear contrast with AAL, which predominated in the stratified cells lining the luminal portion of the duct (Figures 1F and S2). Overall, these data show that the stem cell niches of the limbus, hair follicle, and meibomian gland are associated with reduced cell surface fucosylation.

Fucosylation defines different cell subpopulations in the human limbus

We next sought to investigate the identity of limbal cells with low surface fucosylation on a more granular level. Fluorescence-activated cell sorting (FACS) confirmed that the human limbus, in contrast to central cornea, contained a distinct subset of cells displaying low AAL staining (Figure 2A). This subset of cells, termed AAL^{low}, represented approximately 18% of all the cells in the limbus compared to 3% in central cornea (Figure 2B). In subsequent experiments, and in preparation for single-cell RNA sequencing (scRNA-seq) analysis, we sorted single cells from the limbus into AAL^{low}- and AAL^{high}-binding cell fractions (Figure 2C). We noticed that the addition of AAL caused a marked increase in cell death, as determined by propidium iodide

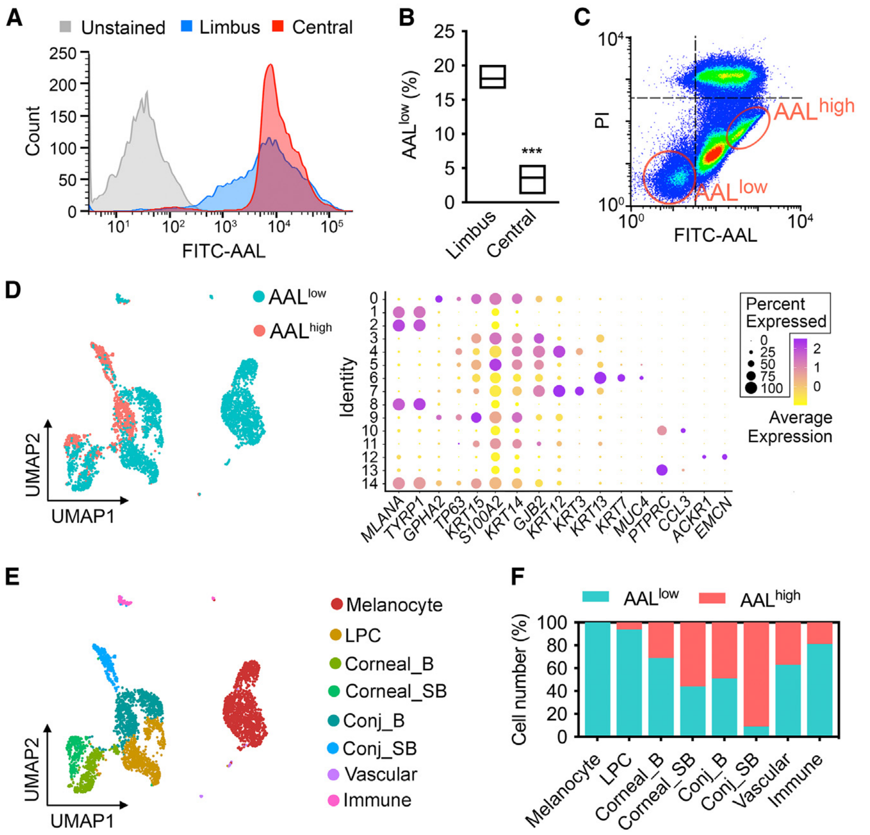

Figure 2. Fucosylation defines different cell subpopulations in the human limbus

(A) Representative FACS histogram showing AAL binding to cell suspensions from the limbus and central cornea.

(B) Box-and-whisker plots showing the percentage of cells displaying low AAL staining (AAL^{low}) in the limbus and central cornea ($n = 3$ human donors; $***p < 0.001$, unpaired t test).

(C) Representative fluorescence-activated cell sorting of limbal cells. All events are plotted in the graph. The red circles define two populations of limbal cells with low and high levels of AAL binding (AAL^{low} and AAL^{high}) collected for scRNA-seq experiments. Propidium iodide (PI) was used to exclude dead cells.

(D) UMAP visualization of AAL^{low} and AAL^{high} cell fractions pooled from limbal tissue of three human donors. The dot plot depicts expression levels of canonical marker genes together with the percentage of cells expressing the marker.

(E) Cell types identified in the human limbus with UMAP projections of scRNA-seq data. LPC, limbal progenitor cell; B, basal; SB, suprabasal; Conj, conjunctival.

(F) Proportions of cell types identified in the AAL^{low} and AAL^{high} cell fractions.

uptake, in cells displaying high lectin staining. This effect has been previously reported and appears to involve the activation of the autophagy pathway (Parshenkov and Hennet, 2022). Consequently, these cells were excluded from the scRNA-seq analysis.

Single-cell transcriptional profiling was performed on sorted AAL^{low} and AAL^{high} cell fractions to identify the types of cells present (Figure 2D). After merging the two datasets and filtering out low-quality cells, the transcriptome profile of 3,180 AAL^{low} and 1,101 AAL^{high} cells was used to create unbiased clusters, which were then visualized by uniform manifold approximation and projection (UMAP). Fourteen clusters were generated by measuring the similarities among transcripts of cells and categorized into cell types based on the expression of canonical marker genes from the literature (Collin et al., 2021). The cell types identified included melanocytes (clusters 1, 2, 8, and 14, expressing *MLANA* and *TYRP1*), limbal progenitor cells (clusters 0 and 9, expressing *GPHA2*, *TP63*, and *KRT15* and the basal markers *S100A2* and *KRT14*), basal corneal epithelial cells (clusters 4 and 11, expressing *KRT12* and the basal marker *GJB2* with relatively low expression of *KRT3*), and suprabasal corneal epithelial cells (cluster 7, expressing both *KRT12* and *KRT3*). There were barely any superficial cells in our analysis, which we attribute to the high fucose content of these cells, which make them more amenable to lectin-induced cell death, and the detachment of the superficial layers of the donor tissue (Figures S3A and S3B) due to routine handling and use of antiseptic measures for ocular surface decontamination. Other cell types identified in our analysis included basal conjunctival epithelial cells (clusters 3 and 5, expressing *KRT13* and *S100A2* with relatively low levels of *KRT7* or *MUC4*), suprabasal conjunctival epithelial cells (cluster 6, expressing *KRT7* and *MUC4* in addition to *KRT13*), immune cells (clusters 10 and 13, expressing *PTPRC* and *CCL3*), and vascular cells (cluster 12, expressing *ACKR1* and *EMCN*).

Altogether, we found eight cell types that were differentially distributed across the AAL^{low} and AAL^{high} datasets (Figure 2E). Quantification of the percentage of cells in each dataset revealed that melanocytes and 94% of limbal progenitor cells displayed low AAL staining, a percentage that was gradually reduced in the more differentiated basal and suprabasal corneal epithelial cells (Figure 2F). Similarly, the percentage of conjunctival epithelial cells in the AAL^{high} group increased with differentiation, with 91% of the suprabasal cells displaying high lectin staining. Immune and vascular cells were distributed across both

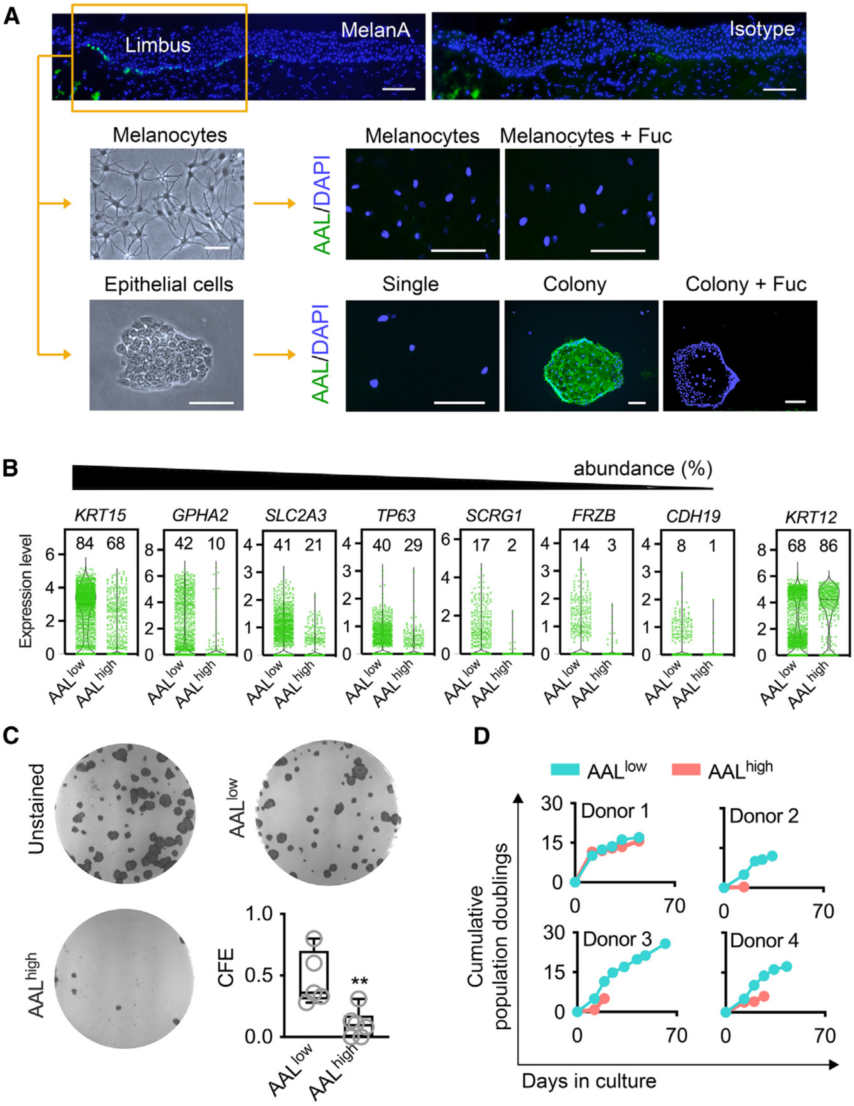

Figure 3. Melanocytes and limbal progenitor epithelial cells display low surface fucosylation

(A) Top: representative micrographs of the human limbus labeled with antibodies to MelanA or isotype control (green). Nuclear DNA was stained with DAPI (blue). Bottom: phase-contrast images of melanocytes and epithelial cells cultured from human limbus. Images to the right show AAL histochemistry (green) and DAPI (blue) staining of non-permeabilized melanocytes and limbal epithelial cells. AAL binding was inhibited in the presence of competing L-fucose (Fuc). Scale bars, 100 μm.

(B) Normalized abundance of epithelial cells expressing limbal progenitor cell markers in AAL^{low} and AAL^{high} cell fractions. Percentages are shown at the top of the scatterplot. Each dot in the scatterplot represents an individual cell. *KRT12* is a marker of cells undergoing corneal epithelial differentiation.

(C) Representative phase-contrast images showing colony-forming ability of AAL^{low} and AAL^{high} cell subpopulations. Unstained cells were processed as control. The box-and-whisker plots show the colony-forming efficiency (CFE) of AAL^{low} and AAL^{high} cells ($n = 5$–6 human donors; $**p < 0.01$, unpaired t test).

(D) Cumulative population doublings of AAL^{low} and AAL^{high} cell fractions obtained from 4 human donors.

datasets. Following the exclusion of non-corneal epithelial cells from the dataset, we conducted a recluster analysis to identify transient amplifying cells. This subpopulation displayed low AAL staining, reminiscent of progenitor cells (Figures S3C and S3D). In summary, these results indicate that the human limbal stem cell niche is a low-fucose microenvironment and suggest that fucose levels in the glycocalyx of progenitor cells increase as they become more differentiated.

Melanocytes and limbal progenitor epithelial cells display low surface fucosylation

We performed *in vitro* experiments to further explore the fucosylation character of melanocytes and limbal epithelial cells isolated from human corneoscleral tissue (Figure 3A). Consistent with the scRNA-seq analysis, we found that AAL bound weakly to primary cultures of human limbal melanocytes. Binding of AAL to limbal epithelial cells, on the other hand, was dependent on the extent of cell proliferation and differentiation. Single epithelial cells in culture exhibited little or no signal when stained with AAL, while the formation of individual cell colonies was associated with increased staining along the plasma membrane of squamous-looking cells.

Upon careful examination of the molecular signatures in the scRNA-seq datasets, we noted that low AAL staining in the limbal epithelial cell subpopulation was associated with high expression of putative limbal stem cell markers (Figure 3B). These genes have been identified in recent studies of the human limbus using single-cell transcriptomics, and included *KRT15*, *GPHA2*, *SLC2A3*, *TP63*, *SCRG1*, *FRZB*, and *CDH19* (Li et al., 2021; Ligocki et al.,

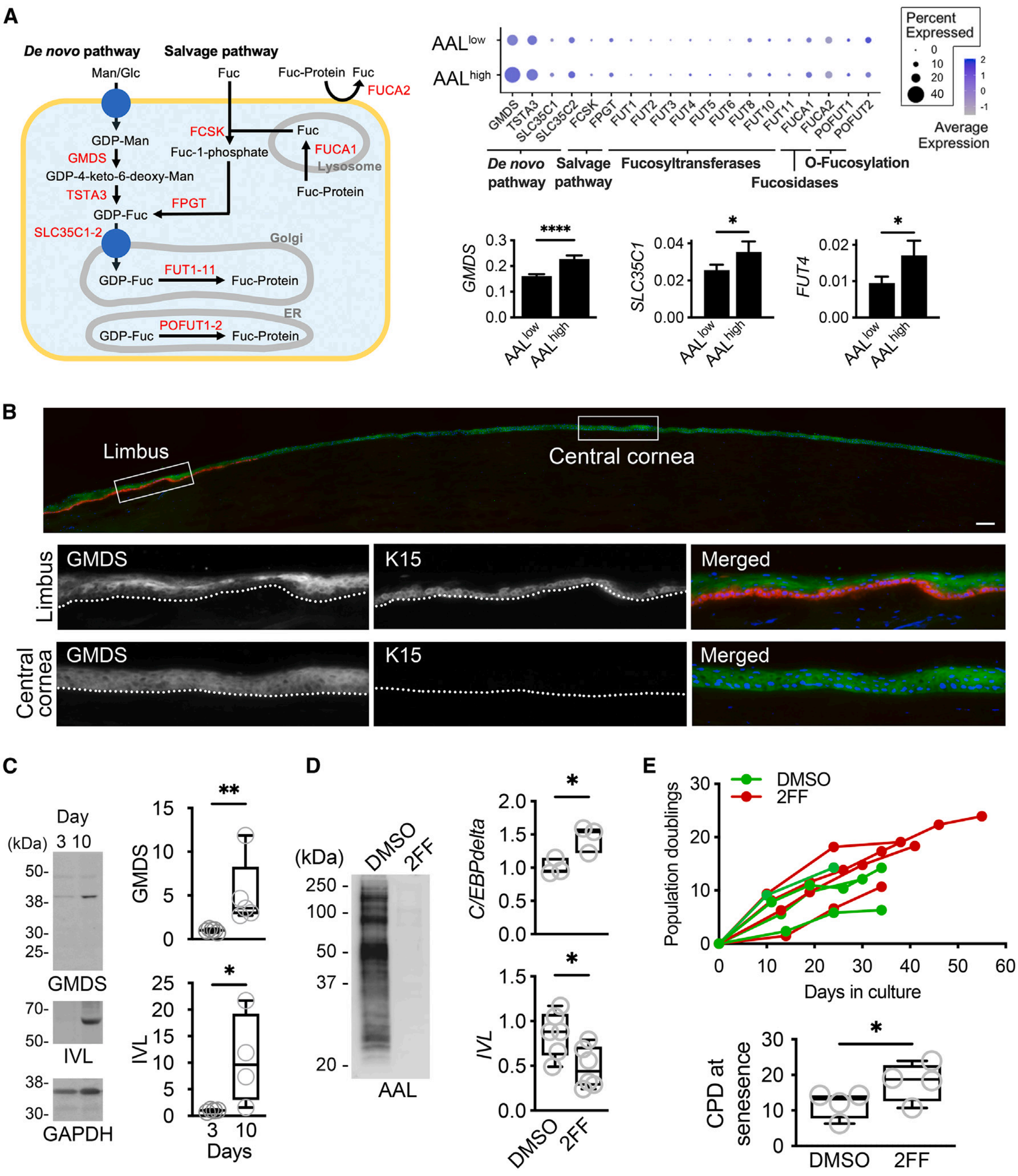

Figure 4. The GDP-fucose *de novo* pathway is hampered in limbal progenitor epithelial cells

(A) Left: schematic overview of the biosynthesis of fucosylated proteins through *de novo* and salvage pathways. Relevant enzymes are labeled in red. Right, top: bubble heatmap showing fucosylation pathway genes in the AAL^{low} and AAL^{high} cell fractions. The size and color of the dot indicate the fraction of expressing cells and averaged scaled expression level for each gene, respectively. Right, bottom: bar graphs depicting genes whose expression is significantly altered between the AAL^{low} and AAL^{high} cell fractions (n = 3 human donors; bar graphs indicate mean ± SEM; $*p < 0.05$, $****p < 0.0001$, Mann-Whitney test).

(B) Representative image of the human cornea co-stained with antibodies to GMDS (green) and K15 (red). Bottom images show magnified images from the same corneal section. Nuclear DNA was stained with DAPI (blue). Dashed lines denote the epithelial-stromal junction. Scale bar, 100 μm.

(C) Immunoblot analysis of human limbal epithelial cells grown for 3 and 10 days. The box-and-whisker plots show the densitometry analysis of the protein band intensity, expressed as the ratio of the target protein/housekeeping protein (n = 4–5 human donors; $*p < 0.05$, $**p < 0.01$, Mann-Whitney test). IVL, involucrin.

(legend continued on next page)

2021; van Zyl et al., 2022). The elevated expression of these markers was observed in a higher percentage of AAL^{low} cells compared to AAL^{high}, with *KRT15*, *GPHA2*, *SLC2A3*, and *TP63* transcripts being present in more than 40% of the AAL^{low} cells. These data prompted us to further investigate whether reduced AAL staining was positively associated with the clonogenic and proliferative capacities of limbal epithelial cells. We found that culture of the AAL^{high}-sorted epithelial cell subpopulation mostly led to aborted, paraclone-type colonies, indicative of replicative senescence (Figure 3C). In contrast, AAL^{low} cells maintained clonogenic capacity as shown by the increased number of colonies present after 10–14 days in culture. Moreover, we found that AAL^{low} cells exhibited a higher number of cumulative population doublings than AAL^{high} cells in three out of four human donor tissues (Figure 3D). Taken together, we find that low fucosylation defines melanocytes and progenitor epithelial cells in the human limbus.

The GDP-fucose *de novo* pathway is hampered in limbal progenitor epithelial cells

Having shown that AAL staining defines limbal epithelial cell subpopulations with divergent clonogenic capacity, we sought to characterize the expression of genes involved in the regulation of fucose synthesis and degradation in each group. As illustrated in Figure 4A, GDP-fucose is produced through two well-identified pathways, the *de novo* pathway and the salvage pathway, and serves as a carbohydrate donor for all fucosyltransferases (Becker and Lowe, 2003). Analysis of the single-cell transcriptomics data revealed that the expression of three genes was significantly lower in the AAL^{low} epithelial cell subpopulation compared to AAL^{high} cells. These included *GMDS*, the first enzyme acting in the *de novo* pathway, and *SLC35C1* and *FUT4*, two enzymes required for the transport of GDP-fucose into the Golgi apparatus and the transfer of α1,3-fucose to N-acetyl glucosamine residues on glycoproteins, respectively (Figure 4A). Furthermore, upon analyzing our datasets in conjunction with those acquired from a public repository, it became evident that progenitor cells displayed reduced expression levels of fucosylation genes when compared to transient amplifying cells (Figures S3E and S3F).

In subsequent experiments, we focused on GMDS since it constitutes a rate-limiting enzyme in the synthesis of GDP-fucose. We asked about the localization of this enzyme in the stratified epithelia of the human cornea and limbus. By immunofluorescence, we found GMDS being present in central cornea and along the suprabasal and superficial cell layers of the human limbus, but not in the basal layer where K15-positive progenitor cells reside (Figure 4B). In concordance with these results, GMDS staining localized toward the more differentiated cells in the hair shaft and was predominant in the stratified cells lining the luminal portion of the meibomian gland duct (Figure S4). These data demonstrate a positive correlation between the cellular distribution of GMDS and AAL and support a role for GMDS in directing the fucosylation of cell surfaces in human stratified epithelia.

Based on these findings, we decided to test the potential of inhibiting fucosylation to enhance the proliferative capacity of primary cultures of human limbal epithelial cells. Toward this end, we used 2F-peracetyl-fucose, an inhibitor of cellular fucosylation that suppresses GMDS activity and causes a reduction in the levels of GDP-fucose (Okeley et al., 2013). We first established that the culture of limbal epithelial cells for up to 10 days led to a significant increase in the relative amount of GMDS, concomitant with an increase in the levels of involucrin—a marker of corneal epithelial cell differentiation (Figure 4C). In subsequent experiments, we showed that the addition of 2F-peracetyl-fucose dramatically reduced protein fucosylation in cultured cells, as shown by AAL lectin blotting (Figure 4D). Remarkably, we observed that culturing limbal epithelial cells with the inhibitor for 7 days resulted in an increased expression of C/EBPdelta, a transcription factor known to influence cell cycle progression (Barbaro et al., 2007), followed by a subsequent decrease in the expression of the differentiation marker involucrin by day 14. Moreover, 2F-peracetyl-fucose led to enhanced cell proliferation capacity, evidenced by a greater number of cumulative population doublings in these cells (Figure 4E). Statistical analysis of the number of doublings at senescence demonstrated that cells incubated with 2F-peracetyl-fucose were able to achieve a better growth rate when compared to the vehicle control, suggesting that manipulation of this pathway could potentially be used therapeutically to promote limbal epithelial cell expansion *ex vivo*.

DISCUSSION

Adult stem cells are present in self-renewing tissues such as the stratified epithelia of the cornea and epidermis, where

(D) Representative lectin blot of human limbal epithelial cells treated with 2F-peracetyl-fucose (2FF) for 14 days. DMSO was used as vehicle control. Right: box-and-whisker plots of qPCR data showing the relative expression of C/EBPdelta and IVL after 7 and 14 days, respectively (n = 3–6 human donors; $^*p < 0.05$, paired t test).

(E) Cumulative population doublings of human limbal epithelial cells treated with 2FF or DMSO (n = 4 human donors). Bottom: box-and-whisker plots showing the cumulative population doublings on senescence (n = 4 human donors; $^*p < 0.05$, unpaired t test).

they replenish dying cells and restore tissue integrity following injury. While there has been significant progress in understanding the biology of these cells, we are only beginning to define how carbohydrates found on their glycocalyx control self-renewal and differentiation. The present study addresses these gaps in knowledge in the human cornea by combining cell sorting of lectin-labeled cells and single-cell transcriptomics. Our results identify the limbal stem cell niche as a low-fucose microenvironment and suggest a role for GMDS in the maintenance of limbal epithelial progenitor cells.

An early study in the *Drosophila* midgut demonstrated that the GDP-fucose *de novo* pathway plays an important role in the self-renewal and commitment of intestinal epithelial stem cells. Loss of the *Drosophila* ortholog for mammalian *GMDS* led to aberrant self-renewing stem cell divisions that generated additional intestinal stem-like cells while simultaneously producing terminally differentiated cells (Perdigoto et al., 2011). The data obtained using this model are compatible with our results showing reduced levels of GMDS in the basal epithelial layer of the limbus, where asymmetric cell division and cell fate decisions occur. The reduced expression of GMDS was also apparent in basal cells of the hair follicle and meibomian gland duct, suggesting that low fucosylation might be an overarching mechanism that influences stem cell fate in human stratified squamous epithelia. It should be noted that, in contrast to these findings, fucose deficiency has been associated with decreased self-renewal in other types of adult stem cells. Deletion of *TSTA3*, an enzyme necessary for the *de novo* synthesis of GDP-fucose, in mouse hematopoietic stem cells led to decreased self-renewal and aberrant hematopoietic phenotypes, as shown by the reduced ability to form multilineage colonies (Myers et al., 2010; Yan et al., 2010). These differences could be related to the distinct functional capacities of stem cells in adult tissues, the individual features of the microenvironment, and the specific signaling pathways that fucose governs in each cell type and species.

The potential consequences of reduced cell surface fucosylation to limbal progenitor cell biology are likely broad and complex. GMDS contributes to regulate the metabolic flux of GDP-fucose through the *de novo* pathway and, consequently, controls the fucosylation of multiple cell surface receptors. For instance, GDP-fucose is necessary for the O-fucosylation of Notch receptors and is also critical to their function (Harvey and Haltiwanger, 2018). Reducing Notch signaling in the human limbus has been shown to decrease the proliferative capacity of epithelial stem cells and maintain the undifferentiated state of limbal stem cells (González et al., 2020; Gonzalez et al., 2019). Consequently, it is expected that low fucose availability in the human limbus will lead to decreased glycosylation of Notch receptors and foster the preservation of limbal stem cell identity. GDP-fucose is also necessary for the biosynthesis of fucosylated Lewis X antigens (Wang et al., 2009). Interestingly, the expression of these fucosylated antigens has been linked to the differentiation stage of corneal epithelial cells in rabbits (Cao et al., 2001), mirroring our histochemistry results showing increased AAL staining in the mature human cornea. Analysis of the expression of fucosyltransferases in rabbit epithelium revealed that *Fut4*, among other fucosyltransferases, supported corneal epithelial cell differentiation by regulating Lewis X-mediated cell-cell interactions (Cao et al., 2001). In our experiments, *FUT4* was one of three genes significantly upregulated in the AAL^{high} epithelial cell subpopulation, suggesting a role for this enzyme in regulating biological functions associated with progenitor cell differentiation in the human limbus.

The culture of limbal epithelial cells for autologous transplantation is becoming an attractive therapeutic modality to treat unilateral limbal stem cell deficiency (Le et al., 2020). Essential to the success of the procedure is the development of new approaches that reliably foster the expansion of epithelial cells from limbal tissue explants and allow for the establishment of a fully functional corneal surface (Jurkunas et al., 2023). Permanent restoration of a transparent, renewing corneal epithelium has been associated with the presence in culture of a high percentage of holoclone-forming stem cells, as defined by the presence of ΔNp63α (Rama et al., 2010). As a result, techniques have been devised to prospectively isolate stem cells from the limbal tissue explants. These techniques have favored the use of protein biomarkers on the cell surface, allowing for monoclonal-antibody-based stem cell sorting strategies (Ksander et al., 2014). Our data showing enhanced clonogenic capacity of cell-sorted AAL^{low} cells indicate that enrichment by negative selection using lectins or carbohydrate-binding reagents is a viable alternative to the use of protein antibodies.

In addition to cell sorting strategies, preserving the glycocalyx of stem cells in culture presents a promising opportunity for therapeutic applications. It is well established that the expansion of limbal epithelial cells in culture is limited by the gradual loss of stemness and proliferative potential over time (Collin et al., 2021; Puri et al., 2022). Our *in vitro* data, which showed a progressive increase in *GMDS* expression and AAL staining over time, prompted us to investigate the potential of inhibiting fucosylation to enhance the proliferative capacity of these cells in culture.

Key insights for this investigation came from previous studies in the fruit fly intestine, which observed that the loss of GMDS in cells that had exited the self-renewing state and become committed was dispensable for terminal

differentiation (Perdigoto et al., 2011). These findings suggested that the success of strategies aimed at expanding limbal epithelium using fucosylation inhibitors would depend on the presence of undifferentiated precursors capable of proliferation and self-renewal.

Efforts to reduce fucosylation by transfecting primary human limbal epithelial cell cultures with *GMDS* small interfering RNA led to decreased GMDS biosynthesis but did not substantially reduce cellular fucosylation. Previous attempts to achieve this had already indicated that near-complete loss of GMDS activity is necessary to substantially diminish fucosylation (Moriwaki et al., 2009). To overcome this limitation, we treated limbal epithelial cells, directly obtained from the cell bank, with 2F-peracetyl-fucose, which resulted in a near-complete inhibition of cell surface fucosylation. This approach enhanced the expression of C/EBPdelta and increased cell proliferation, suggesting the presence of undifferentiated precursors in the early passage population that were responsive to fucose inhibition. This strategy holds promise for future research aimed at improving treatments for limbal stem cell deficiency through the cultivation of epithelial cells.

METHODS

Lectin histochemistry and immunofluorescence

Tissues (see supplemental experimental procedures) were embedded in Tissue-Tek OCT compound (Sakura Finetek), frozen, and sectioned at a thickness of 10 μm. For lectin histochemistry, tissue sections and cell cultures grown on Millicell EZ 8-well glass chamber slides were blocked with 4% bovine serum albumin (BSA) and 2% normal goat serum in Dulbecco's-modified phosphate-buffered saline (DPBS) for 1 h at room temperature before incubation with 1 μg/mL of fluorescein-conjugated *Aleuria Aurantia* lectin (Vector Laboratories) for 2 h at room temperature in the presence or absence of 0.1 M L-fucose. For immunofluorescence detection of MelanA, tissue sections were fixed with 4% paraformaldehyde for 15 min and permeabilized with 0.2% Triton X-100 for 20 min. For detection of GMDS and K15, tissue sections were fixed with ice-cold methanol for 15 min. Sections were blocked with 10% normal goat serum in DPBS for 1 h at room temperature. Then, they were incubated overnight at 4°C with primary antibodies to MelanA (1:1,000, clone EPR20380; Abcam), GMDS (1:50; Proteintech), both GMDS and K15 (1:500, clone LHK15; GeneTex), or isotype controls. Sections were subsequently washed three times with DPBS and incubated with the appropriate secondary antibodies conjugated to Alexa Fluor 488 or 647 (Thermo Fisher Scientific, Southern Biotech) for 1 h at room temperature. Slides were washed with DPBS before addition of FluoroMount-G with DAPI (Thermo Fisher Scientific) and a coverslip. Imaging was performed on a Zeiss Axio Observer Z1 inverted fluorescent microscope (Carl Zeiss Microimaging GmbH).

For lectin-antibody co-staining, human corneal tissue was processed using the AAL staining procedure described earlier followed by permeabilization with 0.2% Triton X-100 for 20 min. Sections were incubated overnight at 4°C with primary antibodies to K15 (1:500, clone LHK15) or ABCG2 (1:100, Bxp-21; Santa Cruz Biotechnology), followed by incubation with the appropriate secondary antibodies conjugated to Alexa Fluor 647 (Thermo Fisher Scientific). For human eyelid tissue, sections were fixed with 4% paraformaldehyde for 15 min and blocked with 10% normal goat serum. Sections were then incubated overnight at 4°C with primary antibodies to K15 (1:400, clone EPR1614Y; Abcam), K5 (1:500; Abcam), or LGR5 (1:50, clone RD42; R&D Systems), followed by the appropriate secondary antibodies and lectin staining.

The regional variation in AAL staining in the corneal epithelium was evaluated by analyzing the images with ImageJ software (National Institutes of Health). A line-intensity scan analysis was performed along the basal layers of the limbus and central cornea. Mean pixel intensity values for the green channel were determined from 5 consecutive, non-overlapping line segments (20 μm long) across each region. Two independent sections from 5 individual donors were used for quantification.

Cell sorting and flow cytometry

Limbal epithelial cell suspensions (see supplemental experimental procedures) were resuspended in flow buffer (2% FBS in DPBS lacking calcium and magnesium and supplemented with 1 mM EDTA). The suspensions were incubated with 1 μg/mL fluorescein-conjugated AAL on ice for 30 min, with gentle mixing every 10 min. Cells were collected by centrifugation, washed with DPBS lacking calcium and magnesium, and resuspended in flow buffer. Immediately before sorting, cells were again filtered with a 35 μm nylon cell strainer. Addition of 3 μM propidium iodide (Thermo Fisher Scientific) was used to exclude dead cells. The stained cells were sorted by a Coulter ELITE cell sorter calibrated for two-color fluorescence. The cells were sorted into two populations, cells with relatively low AAL binding (AAL^{low}) and cells with relatively high AAL binding (AAL^{high}). Flow cytometry was carried out using epithelial cell suspensions from the limbus and central cornea with a BD LSR II flow cytometer (BD Pharmingen).

Colony-forming efficiency and population doubling

To determine colony-forming efficiency, frozen human limbal epithelial cells from the cell bank (see supplemental experimental procedures) were plated onto a 6-well cell culture plate at a density of 1,000 cells/well in the presence of 3T3-J2 fibroblasts and grown as aforementioned with medium changes every 2 to 3 days. When distinct cell colonies were observed in approximately 2 weeks, the colonies were washed, fixed with 4% paraformaldehyde, and stained with 0.2% crystal violet (Sigma-Aldrich) in 10% ethanol. Values are expressed as the ratio of the number of colonies to the number of inoculated cells. For population doubling assays, 1,000 cells from the cell bank were grown on a 12-well cell culture plate in the presence of 3T3-J2 fibroblasts with medium changes every 2 to 3 days. When epithelial cultures reached ~80% confluence, cells were detached, counted, and replated at approximately 1:10 dilution every 10–14 days. Population doubling values were calculated using the following formula: $x = \log_2 N/No$, where N equals the number of epithelial cells harvested at day 14 and No equals the number of seeded cells.

scRNA-seq

Limbal epithelial cell suspensions containing the AAL^{low} or AAL^{high} cell populations were passed through a 35 μm nylon cell strainer, centrifuged for 5 min at 400 × g and washed in 1 mL ice-cold PBS. The cells were centrifuged again for 5 min at 400 × g and resuspended in ice-cold PBS at a ratio of 200 μL PBS per 1 × 10^6 cells, followed by the dropwise addition of ice-cold methanol, at a ratio of 800 μL per 1 × 10^6 cells. The cells were stored at −80°C until sequencing.

Single-cell mRNA sequencing was performed by the Center for Cellular Profiling at Brigham and Women's Hospital. Cells from each condition were resuspended in 0.4% BSA in PBS at a concentration of 1,000 cells per μL. Samples were loaded onto a single lane (Chromium chip, 10× Genomics) and encapsulated in a lipid droplet (Single Cell 3′ kit v.3.1, 10× Genomics). The generation of cDNA and sequencing libraries were performed according to the manufacturer's protocol. The libraries were sequenced to an average of 30,000 reads per cell using Illumina Novaseq.

Processing of the RNA sequencing data

scRNA-seq reads were processed with Cell Ranger v.3.1, which quantified transcript counts per putative cell. Quantification was performed using the STAR aligner against the GRCh38 transcriptome. Data were loaded in R (version 4.2.2) and processed using the Seurat package (version 4.3.0) (Hao et al., 2021). Cells with more than 200 unique molecule identifiers (UMIs) per cell and less than 10% mitochondrial gene transcript content were retained for analysis. The data of both AAL^{low} and AAL^{high} 10X libraries were merged and processed together. The merged data were normalized for sequencing depth per cell and log-transformed using a scaling factor of 10,000. Cells were clustered using graph-based clustering. The differentially expressed genes per cluster were calculated using the Wilcoxon rank-sum test and used to identify cell types.

Immunoblot and lectin blot

Limbal epithelial cells were lysed in radioimmunoprecipitation assay buffer supplemented with complete EDTA-free Protease Inhibitor Cocktail (Roche Diagnostics). After homogenization with a pellet pestle, the cell extracts were centrifuged at 17,115 × g for 30 min at 4°C, and the protein concentration of the supernatants was determined using the Pierce BCA protein assay kit (Thermo Fisher Scientific). Proteins were separated by SDS-PAGE (10% resolving gel) and electroblotted onto nitrocellulose membranes. For immunoblots, membranes were blocked with 5% nonfat milk in 0.1% Tween 20 in Tris-buffered saline for 1 h at room temperature and incubated overnight with primary antibodies to GMDS (1:1,000; Proteintech) or involucrin (1:1,000, SY5; Thermo Fisher Scientific). GAPDH (1:5,000, FL-335; Santa Cruz Biotechnology) staining served as a sample loading control. Membranes were then incubated with the appropriate secondary antibodies conjugated to IRDye 680 or 800 (Li-Cor) for 1 h at room temperature. For lectin blots, membranes were blocked with 1% polyvinylpyrrolidone in Tris-buffered saline–Tween overnight at 4°C. Membranes were then incubated with 1 μg/mL biotin-labeled AAL (Vector Laboratories) for 1.5 h at room temperature. Lectin binding was detected with IRDye 680RD Streptavidin (1:5,000; Li-Cor). Blots were imaged using an Odyssey DLx Imager (Li-Cor). The densitometric analysis of immunoblots was performed with ImageJ software.

qPCR

Total RNA was isolated from epithelial cells using the RNeasy Micro Kit (QIAGEN) following the manufacturer's instructions. Residual genomic DNA in the RNA preparation was eliminated by on-column DNase I digestion (QIAGEN). Total RNA was transcribed using the iScript cDNA Synthesis Kit (Bio-Rad). The qPCR was performed using the SsoAdvanced Universal SYBR Green Supermix (Bio-Rad). Primer sequences for *CEBPD* (Unique Assay ID qHsaCED0048672), *IVL* (Unique Assay ID qHsaCID0008540), and *GAPDH* (Unique Assay ID qHsaCED0038674) mRNA were obtained from Bio-Rad. Gene expression was measured in a Mastercycler ep realplex thermal cycler (Eppendorf) with the following parameters: 2 min at 95°C, followed by 40 cycles of 5 s at 95°C and 30 s at 60°C. Fold changes were calculated using the comparative $\Delta\Delta C_T$ method by normalizing to *GAPDH*.

Statistical analysis

Analysis was performed in GraphPad Prism 7. All *in vitro* experiments were performed in biological replicates of three or more. Values are graphed as bar graphs or box-and-whisker plots showing the 25th and 75th percentiles (boxes), the median, and the minimum and maximum data values (whiskers). Significance was determined using the unpaired or paired t test, or the Mann-Whitney test. Significance was established as $*p < 0.05$.

EXPERIMENTAL PROCEDURES

The procedures describing tissue source, preparation of corneal and limbal epithelial cell suspensions, growth-arrested 3T3-J2 cells, ex vivo expansion and culture of human limbal epithelial cells, and culture of human limbal melanocytes are described in the supplemental experimental procedures.

RESOURCE AVAILABILITY

Lead contact

Further information and requests should be directed to and will be fulfilled or facilitated by the lead contact, Pablo Argüeso (pablo.argueso@tufts.edu).

Materials availability

This study did not generate new unique reagents.

Data and code availability

All the single-cell RNA sequencing data generated in the present study have been deposited at the NCBI Gene Expression Omnibus (GEO: GSE250294) and are publicly available as of the date of publication. No original code and/or algorithms are reported in the present study; however, code used for data analysis can be provided upon request. Any additional information required to reanalyze the data reported in this paper is available from the lead contact upon request.

ACKNOWLEDGMENTS

This work was supported by the National Institutes of Health NEI grant R01EY026147, Massachusetts Lions Eye Research Fund, Inc.; Research to Prevent Blindness, Inc.; and the New England Corneal Transplant Research Fund. We thank Jessica Feldt and Randy Huang for assistance in the technical development of the project.

AUTHOR CONTRIBUTIONS

Conception and design, A.M.W. and P.A.; development of methodology, A.M.W., D.G., and R.M.-C.; acquisition of data, A.M.W. and R.M.-C.; data interpretation, A.M.W., D.G., R.M.-C., E.E.G., R.M.L., and P.A.; writing – reviewing and/or revision of the manuscript, A.M.W., D.G., R.M.-C., E.E.G., R.M.L., and P.A.

DECLARATION OF INTERESTS

P.A. and A.M.W. declare that a provisional patent application related to the methods and findings described in this manuscript has been filed. The provisional patent application is titled "Fucosylation inhibitors for the treatment of ocular diseases" and was submitted on 03/01/2024.

SUPPLEMENTAL INFORMATION

Supplemental information can be found online at https://doi.org/10.1016/j.stemcr.2024.11.008.

Received: January 23, 2024
Revised: November 18, 2024
Accepted: November 19, 2024
Published: December 19, 2024

REFERENCES

Avgustinova, A., and Benitah, S.A. (2016). Epigenetic control of adult stem cell function. Nat. Rev. Mol. Cell Biol. *17*, 643–658. https://doi.org/10.1038/nrm.2016.76.

Bandini, G., Haserick, J.R., Motari, E., Ouologuem, D.T., Lourido, S., Roos, D.S., Costello, C.E., Robbins, P.W., and Samuelson, J. (2016). O-fucosylated glycoproteins form assemblies in close proximity to the nuclear pore complexes of Toxoplasma gondii. Proc. Natl. Acad. Sci. USA *113*, 11567–11572. https://doi.org/10.1073/pnas.1613653113.

Barbaro, V., Testa, A., Di Iorio, E., Mavilio, F., Pellegrini, G., and De Luca, M. (2007). C/EBPdelta regulates cell cycle and self-renewal of human limbal stem cells. J. Cell Biol. *177*, 1037–1049. https://doi.org/10.1083/jcb.200703003.

Becker, D.J., and Lowe, J.B. (2003). Fucose: biosynthesis and biological function in mammals. Glycobiology *13*, 41R–53R. https://doi.org/10.1093/glycob/cwg054.

Bisso, A., Sturla, L., Zanardi, D., De Flora, A., and Tonetti, M. (1999). Structural and enzymatic characterization of human recombinant GDP-D-mannose-4,6-dehydratase. FEBS Lett. *456*, 370–374. https://doi.org/10.1016/s0014-5793(99)00982-5.

Bojar, D., Meche, L., Meng, G., Eng, W., Smith, D.F., Cummings, R.D., and Mahal, L.K. (2022). A Useful Guide to Lectin Binding: Machine-Learning Directed Annotation of 57 Unique Lectin Specificities. ACS Chem. Biol. *17*, 2993–3012. https://doi.org/10.1021/acschembio.1c00689.

Cao, Z., Zhao, Z., Mohan, R., Alroy, J., Stanley, P., and Panjwani, N. (2001). Role of the Lewis(x) glycan determinant in corneal epithelial cell adhesion and differentiation. J. Biol. Chem. *276*, 21714–21723. https://doi.org/10.1074/jbc.M009672200.

Collin, J., Queen, R., Zerti, D., Bojic, S., Dorgau, B., Moyse, N., Molina, M.M., Yang, C., Dey, S., Reynolds, G., et al. (2021). A single cell atlas of human cornea that defines its development, limbal progenitor cells and their interactions with the immune cells. Ocul. Surf. *21*, 279–298. https://doi.org/10.1016/j.jtos.2021.03.010.

Cotsarelis, G., Cheng, S.Z., Dong, G., Sun, T.T., and Lavker, R.M. (1989). Existence of slow-cycling limbal epithelial basal cells that can be preferentially stimulated to proliferate: implications on epithelial stem cells. Cell *57*, 201–209. https://doi.org/10.1016/0092-8674(89)90958-6.

de Morree, A., and Rando, T.A. (2023). Regulation of adult stem cell quiescence and its functions in the maintenance of tissue integrity. Nat. Rev. Mol. Cell Biol. *24*, 334–354. https://doi.org/10.1038/s41580-022-00568-6.

Furukawa, K., Ohkawa, Y., Yamauchi, Y., Hamamura, K., Ohmi, Y., and Furukawa, K. (2012). Fine tuning of cell signals by glycosylation. J. Biochem. *151*, 573–578. https://doi.org/10.1093/jb/mvs043.

Gesteira, T.F., Sun, M., Coulson-Thomas, Y.M., Yamaguchi, Y., Yeh, L.K., Hascall, V., and Coulson-Thomas, V.J. (2017). Hyaluronan Rich Microenvironment in the Limbal Stem Cell Niche Regulates Limbal Stem Cell Differentiation. Invest. Ophthalmol. Vis. Sci. *58*, 4407–4421. https://doi.org/10.1167/iovs.17-22326.

Gonzalez, G., Sasamoto, Y., Ksander, B.R., Frank, M.H., and Frank, N.Y. (2018). Limbal stem cells: identity, developmental origin, and therapeutic potential. Wiley Interdiscip. Rev. Dev. Biol. *7*, e303. https://doi.org/10.1002/wdev.303.

González, S., Halabi, M., Ju, D., Tsai, M., and Deng, S.X. (2020). Role of Jagged1-mediated Notch Signaling Activation in the Differentiation and Stratification of the Human Limbal Epithelium. Cells *9*, 1945. https://doi.org/10.3390/cells9091945.

Gonzalez, S., Uhm, H., and Deng, S.X. (2019). Notch Inhibition Prevents Differentiation of Human Limbal Stem/Progenitor Cells in vitro. Sci. Rep. *9*, 10373. https://doi.org/10.1038/s41598-019-46793-6.

Hao, Y., Hao, S., Andersen-Nissen, E., Mauck, W.M., 3rd, Zheng, S., Butler, A., Lee, M.J., Wilk, A.J., Darby, C., Zager, M., et al. (2021). Integrated analysis of multimodal single-cell data. Cell *184*, 3573–3587.e29. https://doi.org/10.1016/j.cell.2021.04.048.

Harvey, B.M., and Haltiwanger, R.S. (2018). Regulation of Notch Function by O-Glycosylation. Adv. Exp. Med. Biol. *1066*, 59–78. https://doi.org/10.1007/978-3-319-89512-3_4.

Jurkunas, U.V., Yin, J., Johns, L.K., Li, S., Negre, H., Shaw, K.L., Samarakoon, L., Ayala, A.R., Kheirkhah, A., Katikireddy, K., et al. (2023). Cultivated autologous limbal epithelial cell (CALEC) transplantation: Development of manufacturing process and clinical evaluation of feasibility and safety. Sci. Adv. *9*, eadg6470. https://doi.org/10.1126/sciadv.adg6470.

Ksander, B.R., Kolovou, P.E., Wilson, B.J., Saab, K.R., Guo, Q., Ma, J., McGuire, S.P., Gregory, M.S., Vincent, W.J.B., Perez, V.L., et al.

(2014). ABCB5 is a limbal stem cell gene required for corneal development and repair. Nature *511*, 353–357. https://doi.org/10.1038/nature13426.

Lanctot, P.M., Gage, F.H., and Varki, A.P. (2007). The glycans of stem cells. Curr. Opin. Chem. Biol. *11*, 373–380. https://doi.org/10.1016/j.cbpa.2007.05.032.

Le, Q., Chauhan, T., Yung, M., Tseng, C.H., and Deng, S.X. (2020). Outcomes of Limbal Stem Cell Transplant: A Meta-analysis. JAMA Ophthalmol. *138*, 660–670. https://doi.org/10.1001/jamaophthalmol.2020.1120.

Li, D.Q., Kim, S., Li, J.M., Gao, Q., Choi, J., Bian, F., Hu, J., Zhang, Y., Li, J., Lu, R., et al. (2021). Single-cell transcriptomics identifies limbal stem cell population and cell types mapping its differentiation trajectory in limbal basal epithelium of human cornea. Ocul. Surf. *20*, 20–32. https://doi.org/10.1016/j.jtos.2020.12.004.

Ligocki, A.J., Fury, W., Gutierrez, C., Adler, C., Yang, T., Ni, M., Bai, Y., Wei, Y., Lehmann, G.L., and Romano, C. (2021). Molecular characteristics and spatial distribution of adult human corneal cell subtypes. Sci. Rep. *11*, 16323. https://doi.org/10.1038/s41598-021-94933-8.

Lyle, S., Christofidou-Solomidou, M., Liu, Y., Elder, D.E., Albelda, S., and Cotsarelis, G. (1998). The C8/144B monoclonal antibody recognizes cytokeratin 15 and defines the location of human hair follicle stem cells. J. Cell Sci. *111*, 3179–3188. https://doi.org/10.1242/jcs.111.21.3179.

Moriwaki, K., Noda, K., Furukawa, Y., Ohshima, K., Uchiyama, A., Nakagawa, T., Taniguchi, N., Daigo, Y., Nakamura, Y., Hayashi, N., and Miyoshi, E. (2009). Deficiency of GMDS leads to escape from NK cell-mediated tumor surveillance through modulation of TRAIL signaling. Gastroenterology *137*, 188–198. https://doi.org/10.1053/j.gastro.2009.04.002.

Myers, J., Huang, Y., Wei, L., Yan, Q., Huang, A., and Zhou, L. (2010). Fucose-deficient hematopoietic stem cells have decreased self-renewal and aberrant marrow niche occupancy. Transfusion *50*, 2660–2669. https://doi.org/10.1111/j.1537-2995.2010.02745.x.

Okeley, N.M., Alley, S.C., Anderson, M.E., Boursalian, T.E., Burke, P.J., Emmerton, K.M., Jeffrey, S.C., Klussman, K., Law, C.L., Sussman, D., et al. (2013). Development of orally active inhibitors of protein and cellular fucosylation. Proc. Natl. Acad. Sci. USA *110*, 5404–5409. https://doi.org/10.1073/pnas.1222263110.

Parfitt, G.J., Lewis, P.N., Young, R.D., Richardson, A., Lyons, J.G., Di Girolamo, N., and Jester, J.V. (2016). Renewal of the Holocrine Meibomian Glands by Label-Retaining, Unipotent Epithelial Progenitors. Stem Cell Rep. *7*, 399–410. https://doi.org/10.1016/j.stemcr.2016.07.010.

Parshenkov, A., and Hennet, T. (2022). Glycosylation-Dependent Induction of Programmed Cell Death in Murine Adenocarcinoma Cells. Front. Immunol. *13*, 797759. https://doi.org/10.3389/fimmu.2022.797759.

Perdigoto, C.N., Schweisguth, F., and Bardin, A.J. (2011). Distinct levels of Notch activity for commitment and terminal differentiation of stem cells in the adult fly intestine. Development *138*, 4585–4595. https://doi.org/10.1242/dev.065292.

Polkoff, K.M., Gupta, N.K., Green, A.J., Murphy, Y., Chung, J., Gleason, K.L., Simpson, S.G., Walker, D.M., Collins, B., and Piedrahita, J.A. (2022). LGR5 is a conserved marker of hair follicle stem cells in multiple species and is present early and throughout follicle morphogenesis. Sci. Rep. *12*, 9104. https://doi.org/10.1038/s41598-022-13056-w.

Puri, S., Moreno, I.Y., Sun, M., Verma, S., Lin, X., Gesteira, T.F., and Coulson-Thomas, V.J. (2022). Hyaluronan supports the limbal stem cell phenotype during ex vivo culture. Stem Cell Res. Ther. *13*, 384. https://doi.org/10.1186/s13287-022-03084-8.

Rama, P., Matuska, S., Paganoni, G., Spinelli, A., De Luca, M., and Pellegrini, G. (2010). Limbal stem-cell therapy and long-term corneal regeneration. N. Engl. J. Med. *363*, 147–155. https://doi.org/10.1056/NEJMoa0905955.

Rouhanifard, S.H., Lopez Aguilar, A., Meng, L., Moremen, K.W., and Wu, P. (2018). Engineered Glycocalyx Regulates Stem Cell Proliferation in Murine Crypt Organoids. Cell Chem. Biol. *25*, 439–446.e5. https://doi.org/10.1016/j.chembiol.2018.01.010.

Scheper, A.F., Schofield, J., Bohara, R., Ritter, T., and Pandit, A. (2023). Understanding glycosylation: Regulation through the metabolic flux of precursor pathways. Biotechnol. Adv. *67*, 108184. https://doi.org/10.1016/j.biotechadv.2023.108184.

Secker, G.A., and Daniels, J.T. (2009). Limbal epithelial stem cells of the cornea. In StemBook, ed. The Stem Cell Research Community, StemBook. https://doi.org/10.3824/stembook.1.48.1.

Skurska, E., Szulc, B., Maszczak-Seneczko, D., Wiktor, M., Wiertelak, W., Makowiecka, A., and Olczak, M. (2022). Incorporation of fucose into glycans independent of the GDP-fucose transporter SLC35C1 preferentially utilizes salvaged over de novo GDP-fucose. J. Biol. Chem. *298*, 102206. https://doi.org/10.1016/j.jbc.2022.102206.

Sullivan, F.X., Kumar, R., Kriz, R., Stahl, M., Xu, G.Y., Rouse, J., Chang, X.J., Boodhoo, A., Potvin, B., and Cumming, D.A. (1998). Molecular cloning of human GDP-mannose 4,6-dehydratase and reconstitution of GDP-fucose biosynthesis in vitro. J. Biol. Chem. *273*, 8193–8202. https://doi.org/10.1074/jbc.273.14.8193.

Tatapudy, S., Aloisio, F., Barber, D., and Nystul, T. (2017). Cell fate decisions: emerging roles for metabolic signals and cell morphology. EMBO Rep. *18*, 2105–2118. https://doi.org/10.15252/embr.201744816.

van Zyl, T., Yan, W., McAdams, A.M., Monavarfeshani, A., Hageman, G.S., and Sanes, J.R. (2022). Cell atlas of the human ocular anterior segment: Tissue-specific and shared cell types. Proc. Natl. Acad. Sci. USA *119*, e2200914119. https://doi.org/10.1073/pnas.2200914119.

Wang, W., Hu, T., Frantom, P.A., Zheng, T., Gerwe, B., Del Amo, D.S., Garret, S., Seidel, R.D., and Wu, P. (2009). Chemoenzymatic synthesis of GDP-L-fucose and the Lewis X glycan derivatives. Proc. Natl. Acad. Sci. USA *106*, 16096–16101. https://doi.org/10.1073/pnas.0908248106.

Yan, Q., Yao, D., Wei, L.L., Huang, Y., Myers, J., Zhang, L., Xin, W., Shim, J., Man, Y., Petryniak, B., et al. (2010). O-fucose modulates Notch-controlled blood lineage commitment. Am. J. Pathol. *176*, 2921–2934. https://doi.org/10.2353/ajpath.2010.090702.

Stem Cell Reports, Volume *20*

Supplemental Information

Low fucosylation defines the glycocalyx of progenitor cells and melanocytes in the human limbal stem cell niche

Ashley M. Woodward, Damien Guindolet, Rafael Martinez-Carrasco, Eric E. Gabison, Robert M. Lavker, and Pablo Argüeso

Supplemental Figures

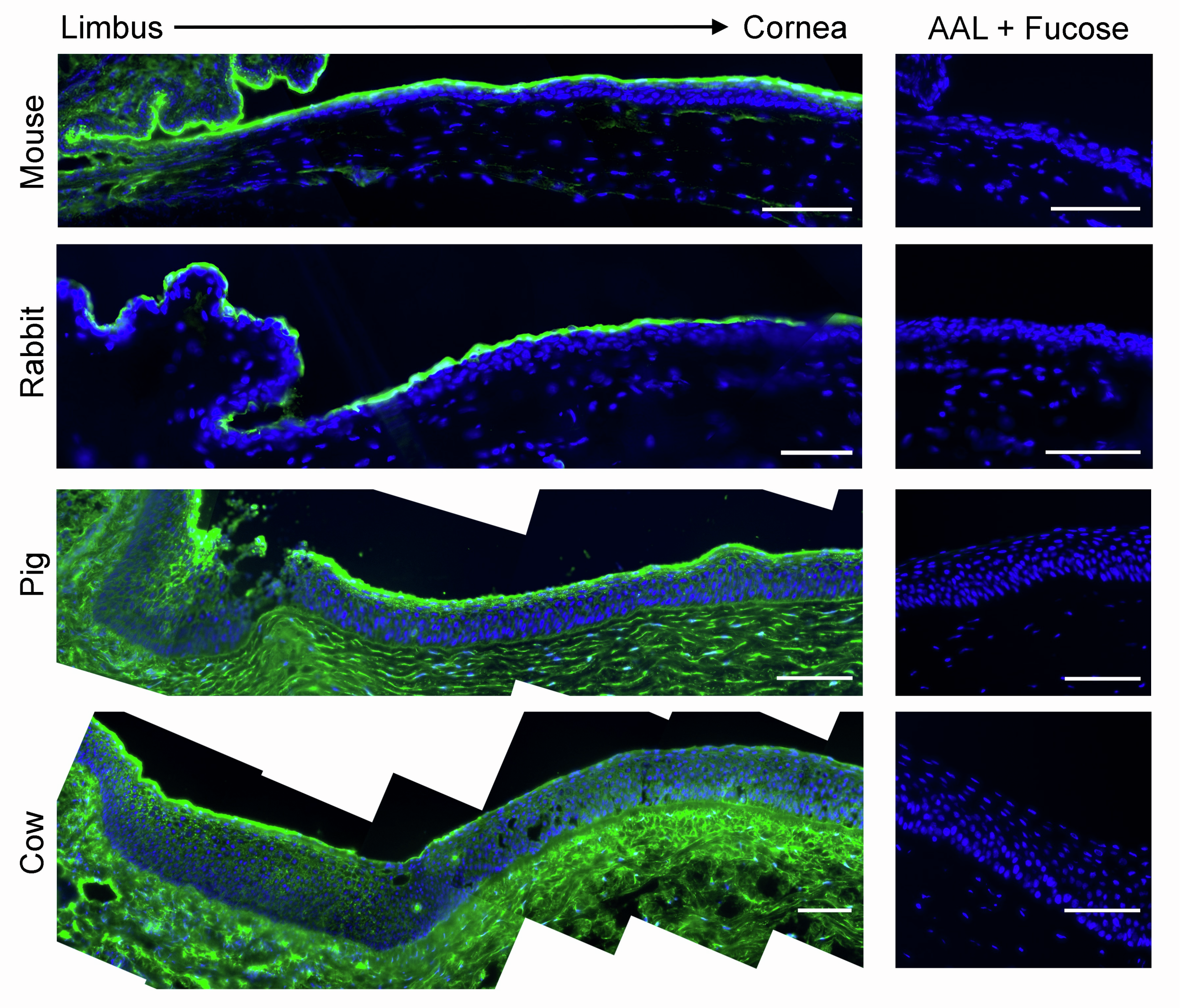

Supplemental Figure S1.

Representative images showing AAL histochemistry (green) and DAPI (blue) in limbus and cornea of different adult mammalian species. AAL binding was inhibited in the presence of competing L-fucose. Scale bars, 100 μm.

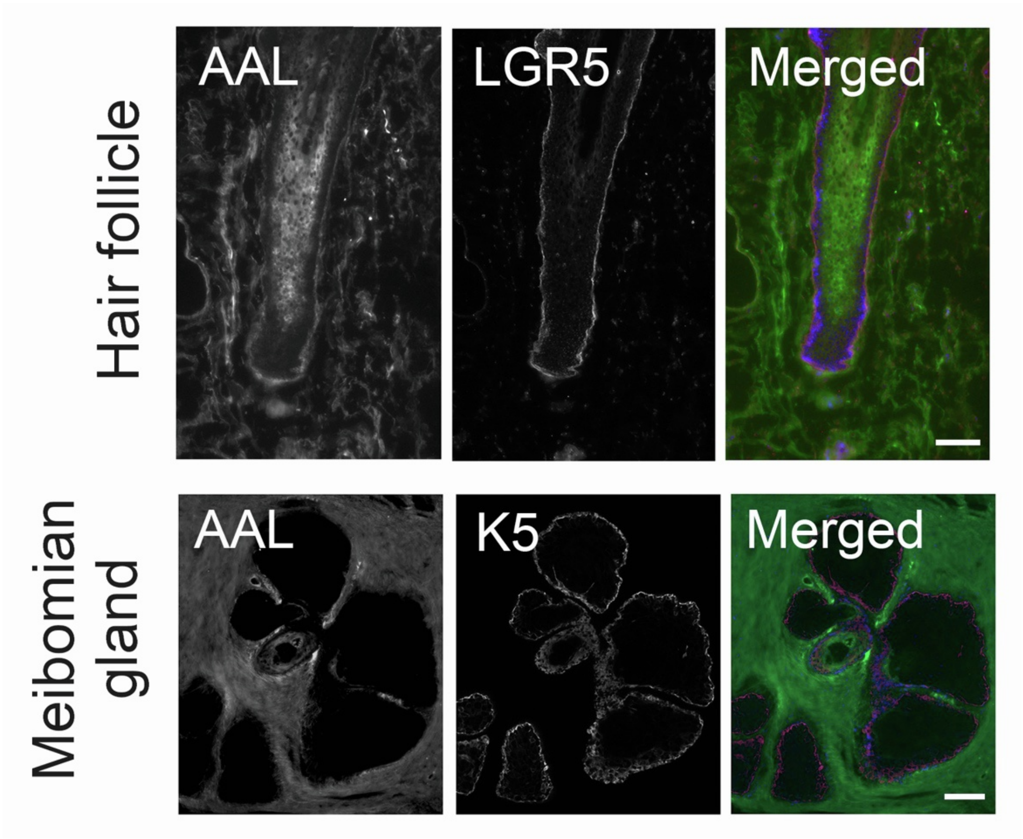

Supplemental Figure S2.

Representative costaining images of AAL (green) and LGR5 (red) in the hair follicle, or cytokeratin 5 (red) in the meibomian gland. Nuclear DNA in the merged image was stained with DAPI (blue). Scale bars, 100 μm.

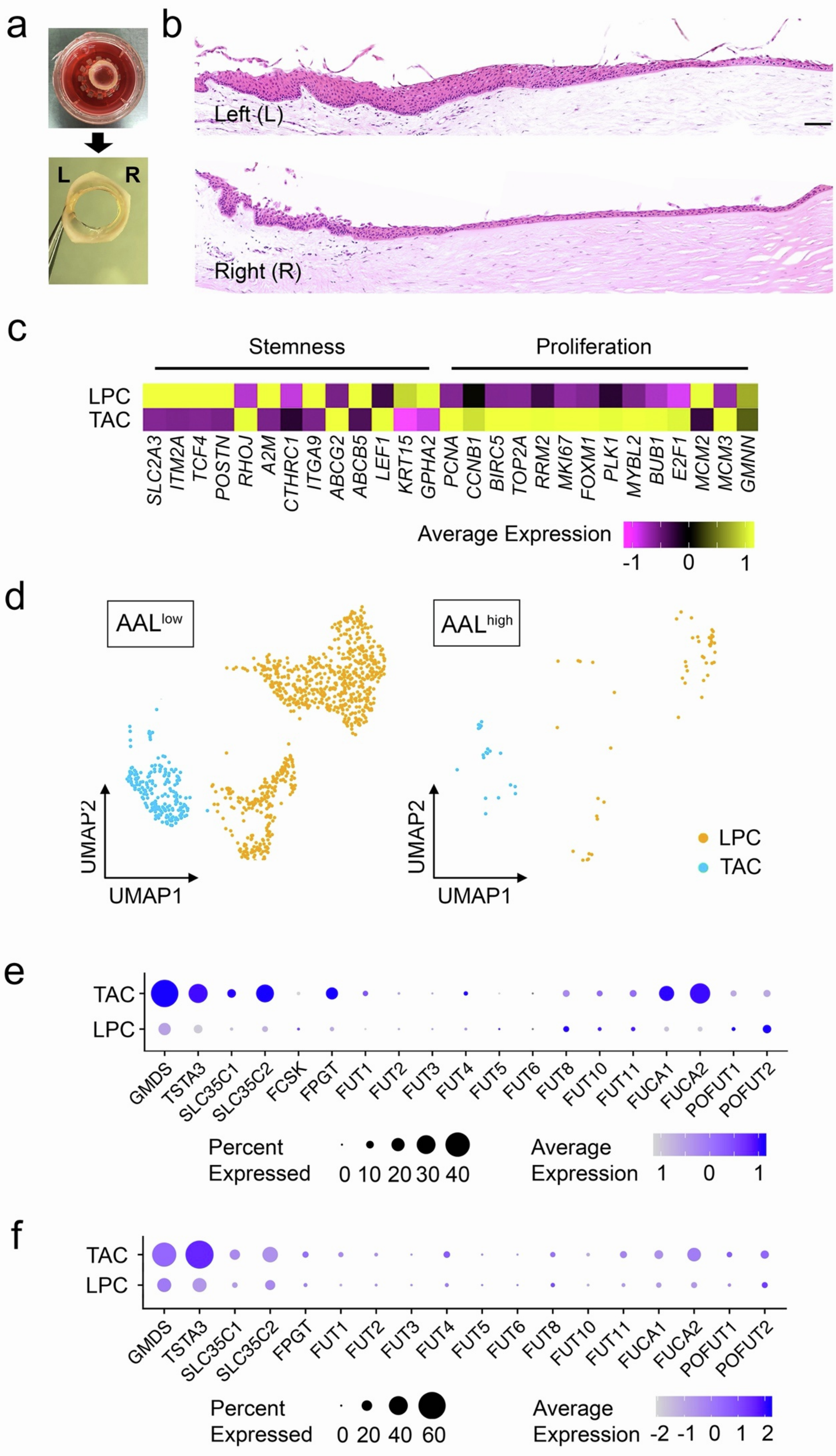

a
b
L
R
Left (L)
Right (R)
c
Stemness
Proliferation
LPC
TAC
SLC2A3
ITM2A
TCF4
POSTN
RHOJ
A2M
CTHRC1
ITGA9
ABCG2
ABCB5
LEF1
KRT15
GPHA2
PCNA
CCNB1
BIRC5
TOP2A
RRM2
MKI67
FOXM1
PLK1
MYBL2
BUB1
E2F1
MCM2
MCM3
GMNN
Average Expression
-1 0 1
d
AAL^low
AAL^high
UMAP2
UMAP1
LPC
TAC
e
TAC
LPC
GMDS
TSTA3
SLC35C1
SLC35C2
FCSK
FPGT
FUT1
FUT2
FUT3
FUT4
FUT5
FUT6
FUT8
FUT10
FUT11
FUCA1
FUCA2
POFUT1
POFUT2
Percent Expressed
0 10 20 30 40
Average Expression
1 0 1
f
TAC
LPC
GMDS
TSTA3
SLC35C1
SLC35C2
FPGT
FUT1
FUT2
FUT3
FUT4
FUT5
FUT6
FUT8
FUT10
FUT11
FUCA1
FUCA2
POFUT1
POFUT2
Percent Expressed
0 20 40 60
Average Expression
-2 -1 0 1 2

Supplemental Figure S3.

(a) Isolation of the limbal region of the cornea. The outer ring contains peripheral cornea, limbus and adjacent conjunctiva. L, left portion; R, right portion.

(b) Histological assessment of peripheral corneal donor tissue (left and right portions) by hematoxylin and eosin staining. Scale bar = 100 μm.

(c) Heatmap showing expression patterns of genes related to stemness and proliferation in limbal progenitor (LPC) and transient amplifying (TAC) cell subpopulations, identified by the reclustering of corneal epithelial cells, as described (Li et al., 2021).

(d) UMAP visualization of LPC and TAC cell subpopulations within the AAL^{low} and AAL^{high} fractions.

(e) The dot plot depicts expression levels of fucosylation genes in the LPC and TAC cell subpopulations together with the percentage of cells expressing each gene in our datasets.

(f) The dot plot depicts expression levels of fucosylation genes in published LPC and TAC cell subpopulations together with the percentage of cells expressing each gene in a database from a public repository (data accessible at NCBI GEO database, accession GSE153515) (Li et al., 2021).

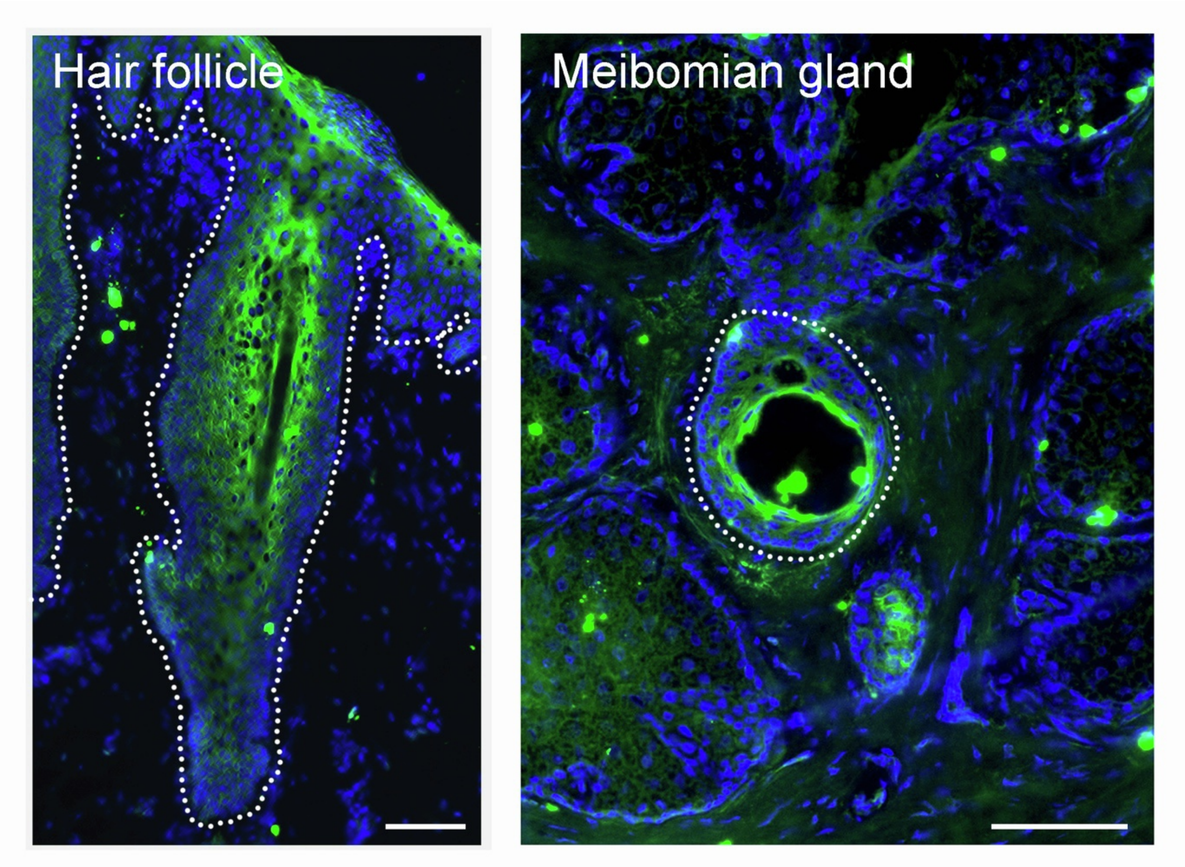

Supplemental Figure S4.

Representative micrographs showing GMDS distribution (green) in human hair follicle and meibomian gland specimens. Nuclear DNA was stained with DAPI (blue). Dashed lines denote the epithelial-stromal junction. Scale bars, 100 μm.

Supplemental Experimental Procedures

Tissue source

All procedures on human tissue were performed in accordance with the Declaration of Helsinki and approved by the Health Sciences Institutional Review Board at Tufts University. Human postmortem corneoscleral tissue unsuitable for transplantation was obtained from Lions VisionGift (Portland, OR) and Saving Sight (Kansas City, MO). Standard eye bank protocols for informed consent and protection of donor confidentiality were used. Standard serologies for infectious agents were negative for all donors. The mean age of the donors (5 females, 5 males) was 61.3 ± 3.8 years (range, 50 to 88 years). The average time from death to preservation of corneoscleral tissue was 14.0 ± 1.4 hours and the average time the tissue was kept in organ culture was 4.6 ± 0.5 days. Archived human eyelid tissue removed during lid resection surgery was obtained from a previous study (Ubels et al., 2012). Eyes from other animal species (cow, pig, rabbit and mouse) were obtained immediately after death from a local slaughterhouse or animal facility.

Preparation of corneal and limbal epithelial cell suspensions

The iris root and endothelium were scraped from corneoscleral tissue using a K-sponge spear (Katena). The central portion of the tissue was cut with an 8-mm disposable biopsy punch (Integra Miltex) to produce corneal rims. Corneal rims and central cornea were subsequently incubated for 1 hour at 37 °C with 2.4 IU/mL Dispase II (Thermo Fisher Scientific) in a supplemented growth medium (sGMedium) containing a 3:1 mixture of DMEM GlutaMAX:F12 with 10% fetal bovine serum, 0.4 μg/mL hydrocortisone, 5 μg/mL insulin, 1.4 ng/mL triiodothyronine, 24 μg/mL adenine, 8.4 ng/mL cholera toxin, 10 ng/mL epidermal growth factor and 1% antibiotic–antimycotic. The epithelia were scraped with a sterile disposable scalpel no. 10 (Integra Miltex), centrifuged at 400 × g for 5 minutes and resuspended in TrypLE Express for another 5 minutes. Cells were then washed with DPBS lacking calcium and magnesium and filtered through a 35 μm nylon cell strainer (Corning).

Growth-arrested 3T3-J2 cells

3T3-J2 mouse fibroblasts (Kerafast) were maintained in DMEM GlutaMAX with 10% bovine calf serum (Hyclone) and 1% penicillin/streptomycin in a humidified incubator with 5% CO_2. Fibroblasts were mitotically inactivated by incubation with 4 μg/mL mitomycin-C (Sigma-Aldrich) for 2 hours at 37 °C. Cells were then rinsed five times with DPBS lacking calcium and magnesium, detached with TrypLE Express (Thermo Fisher Scientific) and frozen in DMEM

GlutaMAX supplemented with 20% bovine calf serum and 10% dimethylsulfoxide (DMSO) for 2 hours at -80 °C before cryopreservation in liquid nitrogen.

Ex vivo expansion and culture of human limbal epithelial cells

Growth-arrested 3T3-J2 fibroblasts were suspended in DMEM GlutaMAX with 10% fetal bovine serum and plated at a density of 4.6 × 10^4 cells/cm^2 in a 100-mm tissue culture dish (Corning). On the following day, limbal epithelial cell suspensions were resuspended in sGMedium and seeded at a density of up to 6 × 10^3 cells/cm^2 in 100-mm tissue culture dishes containing growth-arrested 3T3-J2 fibroblasts. The sGMedium was changed on the third day and every other day thereafter. After 7 days, the 3T3-J2 feeder cells were detached using Versene (Thermo Fisher Scientific) for approximately 1 minute, followed by a wash in DPBS and a 10-minute incubation with TrypLE Express to detach the epithelial cells. These cells were pelleted by centrifugation and frozen (passage 0; cell bank) in DMEM GlutaMAX supplemented with 20% fetal bovine serum and 10% DMSO.

Viable epithelial cells, as determined using Trypan Blue exclusion, were plated at a density of 2 × 10^4 cells/cm^2 for lectin histochemistry or 1 × 10^5 cells/cm^2 for immunoblot and lectin blot. Epithelial cells used for lectin histochemistry, or lectin blot, were grown in the presence of growth-arrested fibroblasts. The fibroblasts were detached for imaging or before protein was collected from the epithelial cells. Immunoblot analyses were performed using protein from epithelial cells grown alone, as previously described (Guindolet et al., 2022). For fucosylation inhibition assays, cells were incubated continuously with 100 μM 2F-peracetyl-fucose (Sigma-Aldrich) or vehicle control (DMSO).

Culture of human limbal melanocytes

Human limbal melanocytes were cultured as previously described (Polisetti et al., 2020). Briefly, 2-10 × 10^5 cells harvested from corneoscleral tissues were seeded onto T75 tissue culture flasks and grown in Medium 254 supplemented with HGMS-2 (Thermo Fisher Scientific). Medium was changed on the first day and every other day thereafter. After 10-14 days, melanocytes and fibroblasts were enzymatically isolated from epithelial cells based on differences in substrate adherence by incubation with a 0.025% trypsin-0.01% EDTA solution (Thermo Fisher Scientific) for 1-3 minutes at 37 °C. After centrifugation, cells were resuspended in Medium 254 supplemented with HGMS-2 and replated at a density of 5 × 10^3 cells/cm^2. Upon reaching 80% confluence, cultures were treated with 0.2 mg/ml geneticin (Thermo Fisher Scientific), an inhibitor of protein synthesis that differentially affects the viability of melanocytes and fibroblasts, for 48 hours to inhibit the growth of fibroblasts. Geneticin was added up to three times to achieve

a yield of >95% melanocytes before expansion in CnT-40 medium (Zen-Bio). Melanocytes were plated onto Millicell EZ 8-well glass chamber slides at a density of 2×10^4 cells/cm^2.

For evaluation of melanocyte enrichment, single cell suspensions of geneticin treated cells were incubated with FcR blocking reagent (20 μl/10^6 cells; Miltenyi Biotec) for 5 minutes and washed with PBS. Cells were then incubated with APC-conjugated anti-CD117 antibody (A3C6E2; Miltenyi Biotec) or isotype control antibody (10 μl/10^6 cells) in PBS containing 0.1% sodium azide and 2% fetal bovine serum for 30 minutes at 4 °C in the dark. After washing with PBS, cells were analyzed with a BD LSR II flow cytometer (BD Pharmingen).

Supplemental References

Guindolet, D., Woodward, A.M., Gabison, E.E., and Argueso, P. (2022). Glycogene expression profile of human limbal epithelial cells with distinct clonogenic potential. Cells *11*. 10.3390/cells11091575.

Li, D.Q., Kim, S., Li, J.M., Gao, Q., Choi, J., Bian, F., Hu, J., Zhang, Y., Li, J., Lu, R., et al. (2021). Single-cell transcriptomics identifies limbal stem cell population and cell types mapping its differentiation trajectory in limbal basal epithelium of human cornea. Ocul Surf *20*, 20-32. 10.1016/j.jtos.2020.12.004.

Polisetti, N., Gießl, A., Li, S., Sorokin, L., Kruse, F.E., and Schlötzer-Schrehardt, U. (2020). Laminin-511-E8 promotes efficient in vitro expansion of human limbal melanocytes. Sci Rep *10*, 11074. 10.1038/s41598-020-68120-0.

Ubels, J.L., Gipson, I.K., Spurr-Michaud, S.J., Tisdale, A.S., Van Dyken, R.E., and Hatton, M.P. (2012). Gene expression in human accessory lacrimal glands of Wolfring. Invest Ophthalmol Vis Sci *53*, 6738-6747. 10.1167/iovs.12-10750.
